# EphrinA2 Regulates Clathrin Mediated KSHV Endocytosis in Fibroblast Cells by Coordinating Integrin-Associated Signaling and c-Cbl Directed Polyubiquitination

**DOI:** 10.1371/journal.ppat.1003510

**Published:** 2013-07-18

**Authors:** Dipanjan Dutta, Sayan Chakraborty, Chirosree Bandyopadhyay, Mohanan Valiya Veettil, Mairaj Ahmed Ansari, Vivek Vikram Singh, Bala Chandran

**Affiliations:** H. M. Bligh Cancer Research Laboratories, Department of Microbiology and Immunology, Chicago Medical School, Rosalind Franklin University of Medicine and Science, North Chicago, Illinois, United States of America; University of North Carolina at Chapel Hill, United States of America

## Abstract

Kaposi's sarcoma-associated herpesvirus (KSHV) interacts with human dermal endothelial cell surface tyrosine kinase EphrinA2 (EphA2) and integrins (α3β1 and αVβ3) in the lipid raft (LR) region, and EphA2 regulates macropinocytic virus entry by coordinating integrin-c-Cbl associated signaling. In contrast, KSHV enters human foreskin fibroblast (HFF) cells by LR-independent clathrin mediated endocytosis. The present studies conducted to identify the key molecules regulating KSHV entry in HFF cells showed that KSHV induces association with integrins (αVβ5, αVβ3 and α3β1) and EphA2 in non-LR regions early during infection and activates EphA2, which in turn associates with phosphorylated c-Cbl, myosin IIA, FAK, Src, and PI3-K, as well as clathrin and its adaptor AP2 and effector Epsin-15 proteins. EphA2 knockdown significantly reduced these signal inductions, virus internalization and gene expression. c-Cbl knockdown ablated the c-Cbl mediated K63 type polyubiquitination of EphA2 and clathrin association with EphA2 and KSHV. Mutations in EphA2's tyrosine kinase domain (TKD) or sterile alpha motif (SAM) abolished its interaction with c-Cbl. Mutations in tyrosine kinase binding (TKB) or RING finger (RF) domains of c-Cbl resulted in very poor association of c-Cbl with EphA2 and decreased EphA2 polyubiquitination. These studies demonstrated the contributions of these domains in EphA2 and c-Cbl association, EphA2 polyubiquitination and virus-EphA2 internalization. Collectively, these results revealed for the first time that EphA2 influences the tyrosine phosphorylation of clathrin, the role of EphA2 in clathrin mediated endocytosis of a virus, and c-Cbl mediated EphA2 polyubiquitination directing KSHV entry in HFF cells via coordinated signal induction and progression of endocytic events, all of which suggest that targeting EphA2 and c-Cbl could block KSHV entry and infection.

## Author Summary

KSHV is etiologically associated with Kaposi's sarcoma and primary effusion B-cell lymphoma. To initiate its *in vitro* infection of endothelial cells, KSHV interacts with cell surface heparan sulfate, integrins, and EphrinA2 (EphA2) molecules in the lipid raft (LR) regions, which induces the integrin-c-Cbl associated signaling and macropinocytic entry. In contrast, KSHV enters human foreskin fibroblast (HFF) cells via LR-independent clathrin mediated endocytosis. The present studies conducted to define the key molecules regulating KSHV entry in HFF cells demonstrate that KSHV induces the association of integrins (αVβ5, αVβ3 and α3β1) with EphA2 in the non-LR regions of HFF cells and activates EphA2, which in turn associates with c-Cbl, myosin IIA, FAK, Src, PI3-K, clathrin, AP2 and Epsin15. Loss of EphA2 function reduces the induction of these signals, virus entry and infection. c-Cbl knockdown also abolishes the EphA2 polyubiquitination and clathrin association with EphA2 and KSHV. These results reveal for the first time the role of EphA2 in clathrin mediated endocytosis of a virus and c-Cbl directed polyubiquitination of EphA2 regulating KSHV infection by coordinating signal induction and underscores EphA2 and c-Cbl as potential targets to intervene in KSHV entry and infection.

## Introduction

During the initiation of infection of target cells, viruses bind to the cellular receptors and utilize a plethora of cellular signal molecules. The utilization of receptors, adaptors and signal molecules largely depends on the nature of the target cells [1]. Animal viruses can utilize different internalization and trafficking pathways that allow specific localization within the cells upon entry for a successful infection. Besides fusion of the viral envelope with the host plasma membrane, receptor mediated endocytosis, an essential biological process mediating cellular internalization events, is often exploited by many enveloped and non-enveloped viruses for their entry into target cells [2], [3]. KSHV, etiologically associated with Kaposi's sarcoma (KS), primary effusion lymphoma (PEL) and multi-centric Castleman's disease (MCD), manifests a wide range of receptor(s) and signal molecules utilization that varies according to the target cell type, serving as an excellent model to determine virus entry associated events [4], [5], [6].

KSHV has a broad range of *in vivo* tropism of target cells such as B, endothelial, epithelial, fibroblast cells, CD34^+^ stem cell precursors of dendritic cells (DCs), monocytes and macrophages [7]. Although KSHV-infected “spindle cells,” are likely of endothelial origin, fibroblast cells are also found in the KS microenvironment, support *de novo* KSHV infection and represent the characteristic component of KS lesions [8]. Following *de novo* infection of skin-derived fibroblasts, KSHV induces the production of pro-inflammatory and pro-migratory factors and promotes endothelial cell invasion of extra cellular matrix (ECM) through paracrine mechanisms [9]. In addition, latent KSHV infection of oral cavity derived primary human fibroblasts enhances the secretion of KS-promoting cytokines and intrinsic invasiveness through VEGF-dependent mechanisms [10], which highlight the potential role for KSHV-infected fibroblasts in promoting KS pathogenesis.

KSHV entry into adherent target cells is a multi-step complex process, involving various viral envelope glycoproteins and multiple cell surface molecules, which overlaps with the induction of pre-existing host signal molecules followed by entry into the cytoplasm, release of viral capsid and transport towards the nucleus via dynein mediated transport along the KSHV induced acetylated thickened bundles of microtubules [6]. KSHV utilizes endocytosis for its entry into human endothelial cells, fibroblasts, B cells and monocytes with different modes of entry depending on cell type [6], [7]. Actin-dependant macropinocytosis and lipid rafts (LRs) are utilized by KSHV to enter human microvascular dermal endothelial (HMVEC-d) cells, while LR-independent clathrin mediated endocytosis is used to enter primary foreskin fibroblast (HFF) cells [6]. Our earlier studies have also demonstrated that during its infection of HMVEC-d and HFF cells, KSHV first binds to cell surface heparan sulfate (HS) molecules and subsequently utilizes integrins α3β1, αVβ3, and αVβ5 [6], [8], [11], [12], [13], [14], [15], [16]. Studies utilizing virus pre-incubated with soluble integrins and anti-integrin antibodies have shown that KSHV interaction with integrin induces a cascade of associated signal molecules such as FAK, Src, PI3-K, RhoGTPases, NF-κB and ERK1/2 which mediates virus endocytic entry, transport towards the nucleus and viral gene expression [6], [8], [11], [12], [13], [14], [15], [16].

Our studies have demonstrated that KSHV macropinocytosis in endothelial (HMVEC-d) cells is controlled by c-Cbl, an adaptor protein, and actin-myosin II A [17]. c-Cbl promoted rapid integrin receptor(s) (α3β1 and αVβ3) translocation into LRs, where monoubiquitination of translocated α3β1 and αVβ3 receptors facilitated KSHV internalization via macropinocytosis, while non-LR bound αVβ5 receptor was polyubiquitinated and targeted towards a non-infectious clathrin mediated pathway to lysosomes [18]. Further studies conducted to determine the potential candidate(s) that coupled KSHV induced integrin signaling with macropinocytosis identified the receptor tyrosine kinase EphA2 as a key molecule mediating KSHV entry [19]. Ephrins have been implicated as a hub for signaling events [20] and ephrin receptors control macropinocytosis and clathrin dependant endocytosis in various cell types [19], [21]. However, the key molecules that regulate KSHV internalization in HFF cells were not characterized. In addition, whether the factor(s) involved in KSHV entry differ based on cell type leading to such varied modes of KSHV entry in endothelial and fibroblast cells is not known.

Studies presented here demonstrate that EphA2 regulates KSHV entry into HFF cells via clathrin mediated endocytosis by coordinating integrin-associated signaling and endocytic events. EphA2 knockdown disrupted KSHV induced signal induction and impaired virus association with clathrin adaptor. Upon KSHV infection c-Cbl ubiquitin ligase with its tyrosine kinase binding (TKB) and RING domains facilitated the interaction with the tyrosine kinase (TK) and sterile alpha motif (SAM) domains of EphA2, thereby promoting EphA2 polyubiquitination (K63 type) critical for clathrin mediated internalization of associated virus. These studies provide the first important insight into understanding the c-Cbl mediated modulation of EphA2 regulating clathrin mediated virus entry and productive infection.

## Results

### EphA2 knockdown inhibits KSHV entry and infection in HFF cells

**Figure 1:**
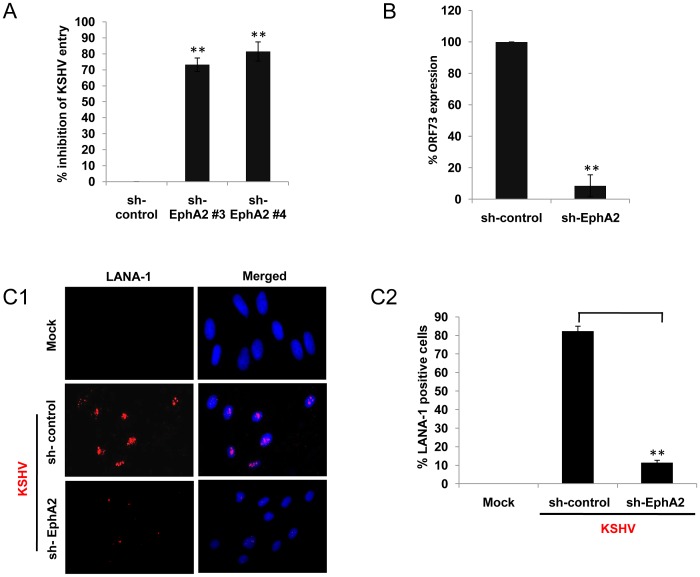
Effect of EphA2 knockdown on KSHV entry and infection in HFF cells. (**A**) Control or different EphA2 shRNA-transduced HFF cells were infected with KSHV (30 DNA copies/cell) for 10 min at 37°C. After washing, total DNA was isolated and virus entry was determined by real-time DNA PCR for KSHV ORF73 gene. Results are presented as percentage of inhibition of KSHV DNA internalization by sh-EphA2 transduced compared to control shRNA transduced HFF cells incubated with virus. Data shown are means ± SD (n = 3; ** statistical significance, P<0.01). (**B**) Control or sh-EphA2 transduced (no 4) HFF cells were infected with KSHV for 2 h. At 48 h.p.i., cells were harvested for RNA isolation and viral gene expression was determined by real-time RT-PCR with KSHV ORF73 gene specific primers. Data represented are means ± SD (n = 3; P<0.01). (**C1 and C2**) Control or sh-EphA2 transduced HFF cells were either mock infected or infected with KSHV (30 DNA copies/cell) for 2 h at 37°C, washed and cultured in complete media for another 46 h. After washing, cells were fixed and processed for immunofluorescence using rabbit anti-LANA-1 antibody. Representative images are shown. The percentage of cells showing punctate LANA -1 dots is represented in the graphical plot. A minimum of 3 fields having at least 20 cells were chosen. Error bars show means ± SD.

Since endothelial and fibroblast cells harbor latent KSHV infection and represent cellular components of KS, it is relevant to characterize the entry components in these model systems [22], [23]. Earlier studies revealed the involvement of EphA2 as one of the important entry receptors for KSHV in endothelial and epithelial cells [19], [24]. To establish a potential role of EphA2 related events during KSHV infection of primary fibroblast cells, we first analyzed whether EphA2 lentivirus-encoding shRNAs which were shown earlier to be effective for maximal EphA2 knockdown [19] could inhibit viral entry. Compared to control shRNA transduced cells, HFF cells transduced with EphA2 shRNA #3 and #4 showed significant reduction in KSHV entry (∼70% and ∼80%, respectively) as determined by measuring the internalized KSHV viral (ORF73) DNA copies (Figure 1A). The greater efficiency of EphA2 shRNA #4 led us to use this shRNA in our subsequent studies.

We next determined the effect of EphA2 shRNA on KSHV latent gene expression in HFF cells since KSHV latency establishment in these cells represents successful virus infection [25]. Control shRNA or EphA2 shRNA-transduced HFF cells were infected with KSHV for 48 h and viral latent gene expression was determined by real-time RT-PCR for the latency associated ORF73 (LANA-1) gene. Compared to control shRNA transduced cells, HFF cells transduced with EphA2 shRNA showed ≥90% inhibition of ORF73 gene expression (Figure 1B). In conjunction with this we also observed ≥80% reduction in the punctate nuclear staining of LANA-1 at 48 h post infection (p.i.) in EphA2 shRNA transduced HFF cells compared to control sh-RNA cells (Figure 1, C1 and C2). Taken together, these results suggested that inhibition of viral gene expression in EphA2 knockdown cells was most likely due to blocking entry into the fibroblast cells, and EphA2 plays a significant role in KSHV entry.

### KSHV infection induces activation and association of EphA2 with virus particles and entry associated integrin receptors

**Figure 2:**
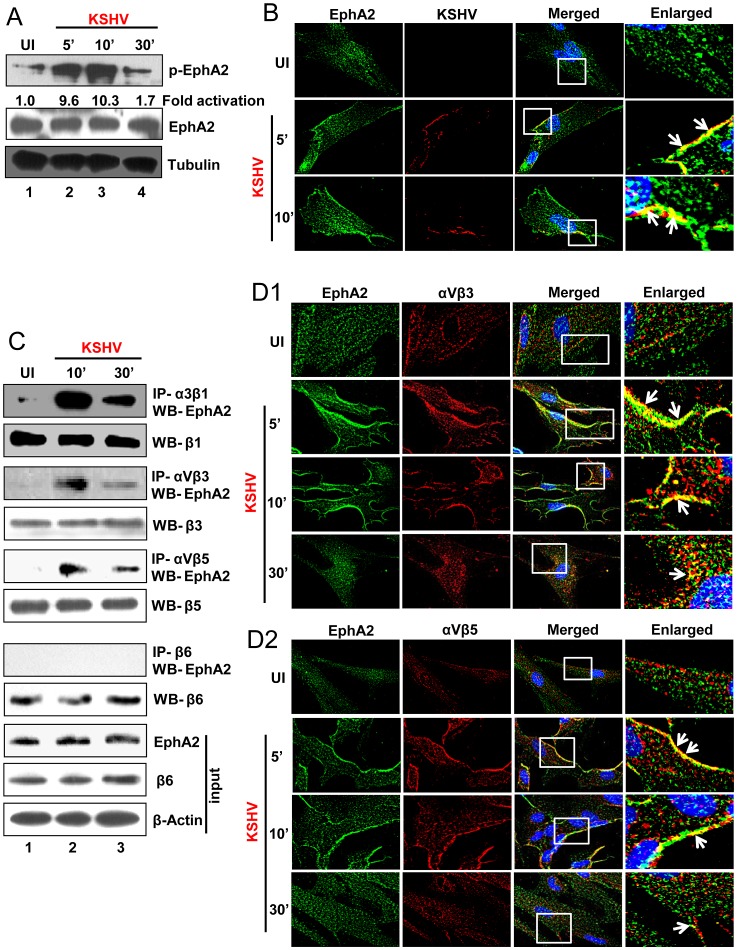
EphA2 is activated and associated with entry receptors early during KSHV infection. (**A**) Serum-starved (8 h) HFF cells were either mock infected (UI) or infected with KSHV (30 DNA copies/cell) for the indicated time periods and subjected to Western blot analysis for phospho-EphA2 (Y594) (pEphA2). The blot was stripped and reprobed for total EphA2 and tubulin was used as loading control. (**B**) Serum starved HFF cells were either mock infected or infected with KSHV (30 DNA copies/cell) for 5 and 10 min and processed for immunofluorescence analysis using rabbit anti-EphA2 and mouse monoclonal anti-KSHV gpK8.1A antibodies for overnight at 4°C followed by staining with anti-rabbit Alexa 488 and anti-mouse Alexa 594 secondary antibodies. Representative 2D convoluted images are shown. The white boxes within the merged panels are shown as enlarged pictures and the white arrows represent colocalization of the indicated molecules. (**C**) Serum-starved HFF cells were left uninfected or infected with KSHV for the indicated periods of time and immunoprecipitated with anti-α3β1, αVβ3 or αVβ5 antibodies and analyzed for EphA2 by Western blot (first, third and fifth panels). These blots were stripped and reprobed for total β1, β3 and β5 integrin subunits, respectively (second, fourth and sixth panels). For negative control, cell lysates from uninfected or KSHV infected HFF cells were immunoprecipitated with anti-β6 antibody and analyzed for EphA2 by western blotting (seventh panel). The blot was stripped and reprobed with total β6 antibodies (eighth panel). Whole cell lysates were subjected to western blot analysis for input expression of EphA2 (ninth panel) and β6 (tenth panel) and β-actin was used as loading control (eleventh panel). (**D**) Serum starved HFF cells were either left uninfected or infected with KSHV (30 DNA copies/cell) for 5, 10 and 30 min, washed and processed for immunofluorescence analysis. Cells were permeabilized, blocked with blocking reagents and incubated with (**D1**) rabbit anti-EphA2 and mouse anti-αVβ3 antibodies or (**D2**) rabbit anti-EphA2 and mouse anti-αVβ5 antibodies for 2 h at room temperature followed by staining with anti-rabbit Alexa fluor 488 or anti-mouse Alexa fluor 594. Representative 2D deconvoluted images are shown. The enlarged pictures represent boxed regions within the merged panels and the white arrows represent colocalization of the indicated molecules.

EphA2, a receptor tyrosine kinase (RTK), is functionally activated through tyrosine phosphorylation essential for receptor signaling and internalization [26], [27]. Since previous studies demonstrated that KSHV infection induced EphA2 phosphorylation in endothelial and epithelial cells [19], [24] we determined the activation of EphA2 during primary KSHV infection of HFF cells. As shown in Figure 2A, EphA2 was phosphorylated as early as 5 min p.i., increased at 10 min p.i. and reduced by 30 min p.i.. At 5 min and 10 min p.i, colocalization between EphA2 and KSHV (glycoprotein gpK8.1A) was observed in virus infected HFF cells compared to mock infected (Un) control (Figure 2B). Since EphA2 interacted with integrin receptors during KSHV infection in endothelial cells [19], we determined their associations in KSHV infected fibroblast cells through coimmunoprecipitation. We observed considerable interactions of EphA2 with integrins α3β1, αVβ3 or αVβ5 in KSHV infected cells at 10 min p.i. which decreased by 30 min p.i. (Figure 2C, first, third and fifth panels). However, there was no interaction between EphA2 and integrin β6 in virus infected HFF cells (Figure 2B, seventh panel) indicating KSHV induced specific association of those integrin molecules with EphA2. Immunofluorescence analyses further supported these findings where we observed significant colocalizations of integrins αVβ3 or αVβ5 with EphA2 at the initial time of infection which were more pronounced at the peripheral regions of KSHV infected cell (Figure 2, D1 and D2; white arrows). We also observed similar colocalization between EphA2 and α3β1 intergrin (data not shown). These results demonstrated that early during HFF cell infection, KSHV induces the activation of EphA2 which interacts with virus particles and entry associated integrin receptors.

### EphA2 associates with effector(s) critical for clathrin mediated endocytosis during KSHV infection of HFF cells

**Figure 3:**
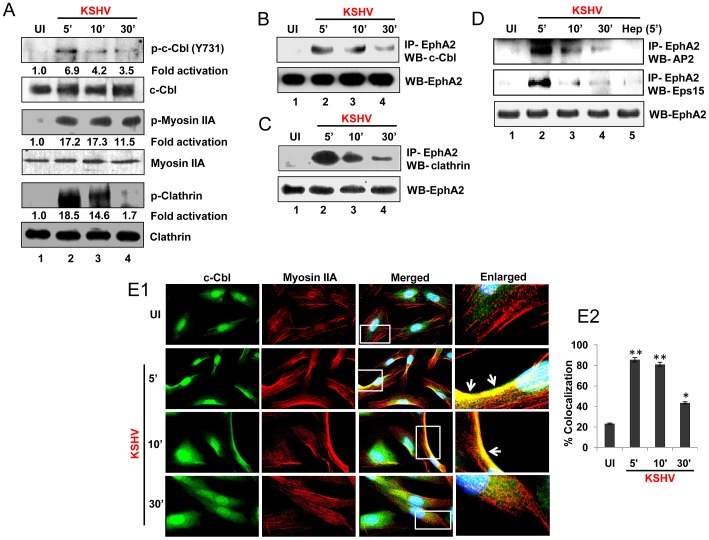
Endocytic effectors critical for clathrin mediated entry are activated and associated with EphA2 early during KSHV infection. (**A**) Serum-starved HFF cells were either mock infected (UI) or infected with KSHV (30 DNA copies/cell) for the indicated time periods and subjected to Western blot analysis for phospho-c-Cbl (p-c-Cbl) (Y731) and phospho-myosin IIA (S-19). For clathrin activation, cell lysates were subjected to immunoprecipitation with anti-clathrin heavy chain antibody followed by Western blot analysis with anti-phosphotyrosine antibody (4G10). Blots were stripped and reprobed for the respective total c-Cbl, myosin and clathrin. (**B,C and D**) Serum-starved HFF cells were left uninfected (UI) or infected with KSHV for the indicated periods of time and immunoprecipitated with anti-EphA2 antibodies and analyzed for c-Cbl, clathrin or clathrin adaptors AP2 and Epsin15 by Western blots which were stripped and reprobed for EphA2. For heparin treatment of KSHV, virus was pre-incubated with heparin (100 µg/ml) for 1 h at 37°C and used for infecting cells for 5 min. (**E**) Serum-starved HFF cells either uninfected or infected with KSHV for the indicated time points were subjected to immunofluorescence assay using mouse anti-c-Cbl and rabbit anti-myosin IIA antibodies for 2 h at room temperature followed by staining with anti-mouse Alexa fluor 488 and anti-rabbit Alexa fluor 594, respectively. Representative 2D convoluted images are shown (**E1**). The enlarged pictures represent boxed regions within the merged panels and the white arrows represent colocalization of the indicated molecules. Quantitation of cells showing c-Cbl and myosin IIA colocalization (**E2**). At least three different microscopic fields having at least 10 cells each were chosen to analyze the colocalization efficiency. Error bars represent mean ± SD, * represents p<0.05 and ** represents p<0.01.

Our earlier studies have shown that KSHV utilizes clathrin dependant endocytosis to enter HFF cells without the involvement of lipid rafts [8], [22]. However, the factors involved in clathrin-mediated internalization of KSHV were not determined. Since we observed the activation and association of EphA2 with virus particles and KSHV entry receptors, we hypothesized that KSHV-induced EphA2 could be modulating this internalization pathway in HFF cells. To test this hypothesis, we first identified the activities of major endocytic factors required for KSHV entry in HFF cells based on our earlier studies that c-Cbl, myosin IIA and clathrin are implicated in endocytosis of KSHV in various cell types [17], [18], [22]. Following virus infection, a 3- to 7-fold increase in c-Cbl phosphorylation and 11- to 17- fold increase in p-myosin IIA was observed at 5, 10, and 30 min p.i., whereas a 14- to 18-fold increase in phosphorylation of clathrin heavy chain was observed at 5 and 10 min which was subsequently decreased at 30 min p.i. (Figure 3A). There was no significant change in basal levels of c-Cbl, myosin IIA or clathrin which demonstrated that KSHV modulates activation of these entry effectors.

Association of KSHV entry receptor integrins with EphA2 and activation of virus induced endocytic effectors led us to investigate whether EphA2 could also associate with those effector molecules essential for clathrin mediated virus entry. Lysates from mock infected HFF cells or infected with KSHV for 5–30 min were immunoprecipitated with anti-EphA2 antibody followed by Western blotting for c-Cbl, clathrin and clathrin adaptors AP2 and Epsin15. In contrast to uninfected cells, KSHV infection increased the association of EphA2 with c-Cbl, clathrin, AP2 and Epsin15, which maximized within 5 min p.i. and subsequently reduced by 10 and 30 min p.i. (Figures 3, C and D). When cells were infected for 5 min with KSHV pre-incubated with heparin (100 µg/ml), we observed the near absence of AP2 and Epsin15 interactions with EphA2 (Figure 3D), which not only demonstrated the specificity but also suggested that KSHV infection induced association of EphA2 with endocytic effectors. Compared to uninfected cells we observed substantial colocalization of c-Cbl and myosin IIA in KSHV infected HFF cells from 5 and 10 min p.i., which decreased by 30 min p.i. (Figure 3E1). This result demonstrated that this is a virus infection induced event. With respect to uninfected cells mean pixel intensities of colocalization between c-Cbl and myosin IIA were significantly higher in KSHV infected HFF cells at 5 and 10 min p.i. (≥81%) which was less increased at 30 min p.i. (≥42%) (Figure 3E2).

**Figure 4:**
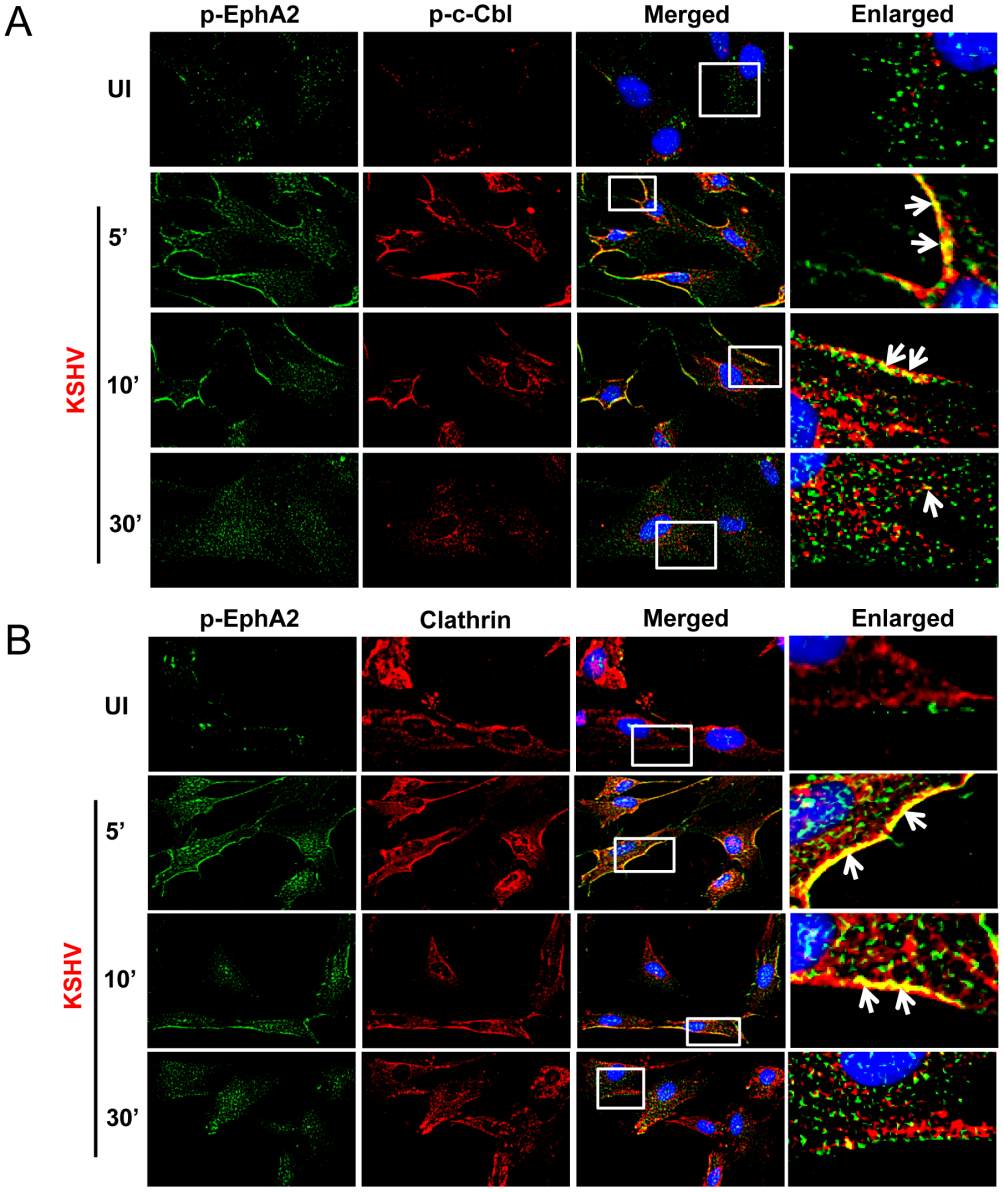
Activated EphA2 colocalizes with activated c-Cbl and clathrin. Serum-starved HFF cells either uninfected or infected with KSHV (30 DNA copies/cell) for the indicated time points were subjected to immunofluorescence assay using rabbit anti-phospho-EphA2 (p-EphA2) and (**A**) mouse anti-phospho-c-Cbl or (**B**) clathrin heavy chain antibodies for 2 h at room temperature followed by staining with anti-rabbit Alexa fluor 488 and anti-mouse Alexa fluor 594, respectively. Representative 2D convoluted images are shown. The enlarged pictures represent boxed regions within the merged panels and the white arrows represent colocalization of the indicated molecules.

Efficient tyrosine phosphorylation of EphA2, c-Cbl and clathrin and the presence of c-Cbl and clathrin with EphA2 co-immunoprecipitates (Figures 2A, 3B and 3C) led us to further determine whether phosphorylated EphA2 associates with activated effector molecules. Compared to mock infection, early during KSHV infection (5 and 10 min p.i.), a significant colocalization was observed between p-EphA2 and p-c-Cbl or clathrin predominantly at the periphery of infected HFF cells (Figures 4A and 4B, white arrows) with concomitant reduction of association at 30 min p.i. (Figures 4A and 4B).

Taken together these studies suggested that c-Cbl, myosin IIA, and clathrin with its adaptor molecules AP2 and Epsin15 are recruited by activated EphA2 to form a KSHV-macromolecular internalization complex essential for clathrin mediated virus entry.

### EphA2 predominantly localizes and associates with integrins and clathrin to the Non-Lipid-Raft (NLR) fractions of KSHV infected HFF cells

**Figure 5:**
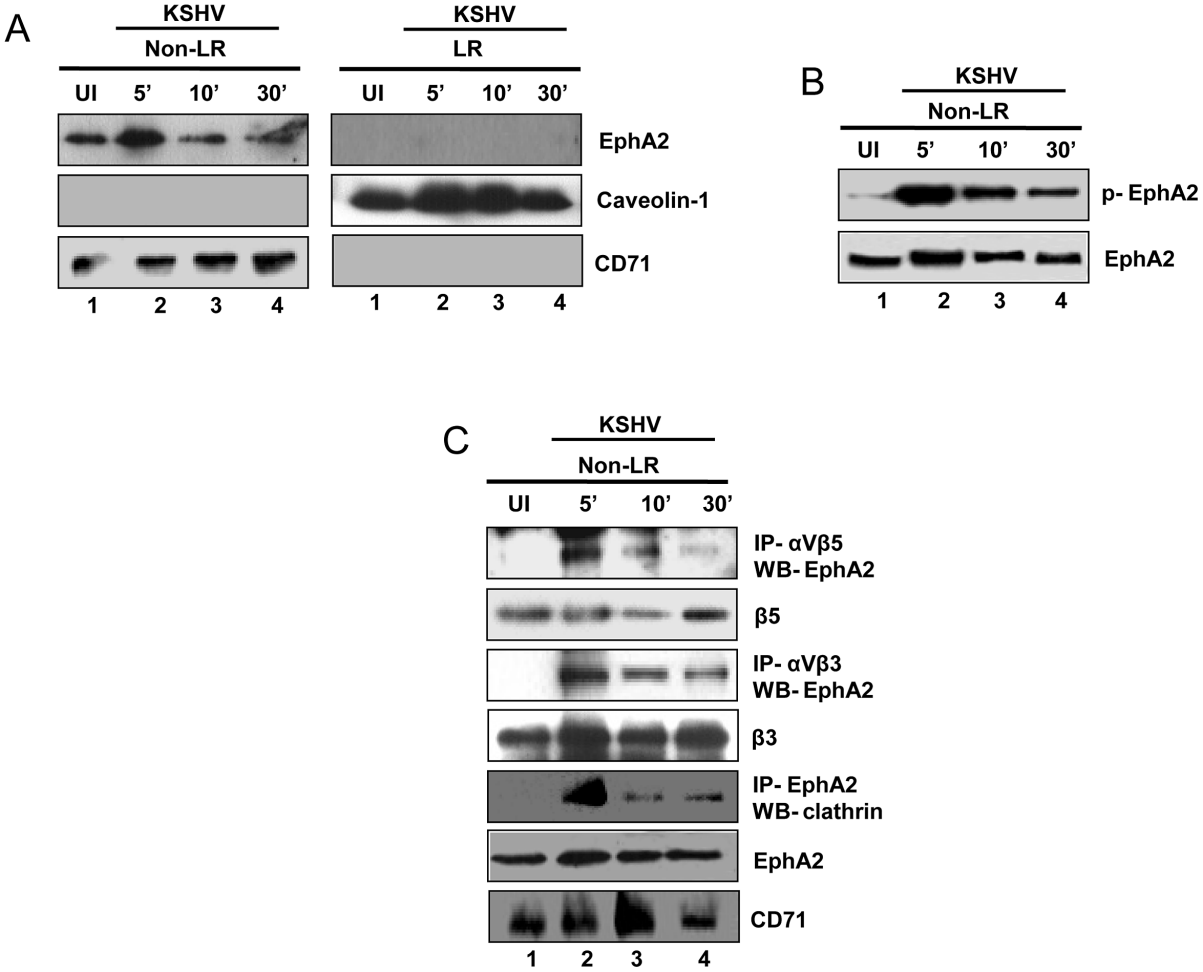
EphA2 associated with integrins and clathrin are localized to non-lipid rafts in KSHV infected HFF cells. (**A**) Serum-starved HFF cells were either uninfected or infected with KSHV (30 DNA copies/cell) for the indicated time points. LR and non-LR fractions were isolated and analyzed by Western blotting for the presence of EphA2. Caveolin-1 and CD71 characterize the purity of LR and non-LR fractions, respectively. (**B**) Western immunoblot analysis showing phosphorylation of EphA2 at the indicated time post-KSHV infection in non-NLR fractions from HFF cells. (**C**) 150 µg of protein from non-LR fractions of uninfected and KSHV-infected cells was immunoprecipitated with anti- αVβ5 or anti-EphA2 antibodies at 4°C overnight followed by Western blotting with EphA2 or clathrin heavy chain, respectively. Blots were stripped and reprobed for β5 and β3, respectively. CD71 characterizes the purity of non-LR fractions.

The above results demonstrated the association and colocalization of EphA2 with KSHV entry receptors and endocytic effectors preferentially at the periphery of HFF cells early during infection. Together with previous studies which suggested that KSHV enters into HFF cells via clathrin mediated endocytosis without involving lipid rafts [22] prompted us to next assess the localization of EphA2 in KSHV infected HFF cells. In uninfected and virus infected cells, EphA2 was predominately located in the NLR portions of plasma membranes (Figure 5A). In addition, compared to mock-infected NLR fractions, we observed about a 7-fold increase in EphA2 phosphorylation at 5 min p.i. which gradually decreased by 10 min and 30 min p.i. (Figure 5B). To further confirm the significance of EphA2 in NLR associated KSHV entry, NLR fractions prepared from mock or KSHV infected HFF cells at 5, 10 and 30 min p.i. were immunoprecipitated (IP-ed) with anti-αVβ5 or -αVβ3 antibodies. Western blotting of the IP-fractions for EphA2 demonstrated a strong association of EphA2 with these integrins at 5 min p.i. which gradually decreased by 10 or 30 min p.i. (Figure 5C, first and third panel). Interestingly, a similar pattern of interaction was also observed between EphA2 and clathrin from NLR fractions of infected HFF cells (Figure 5C, fifth panel). These results corroborated our early evidences in Figures 3C and 4B and suggested that EphA2 mediated KSHV entry in HFF is a non-LR associated event.

### EphA2 recruits a KSHV-induced FAK, Src and PI3-K signaling complex critical for virus entry

**Figure 6:**
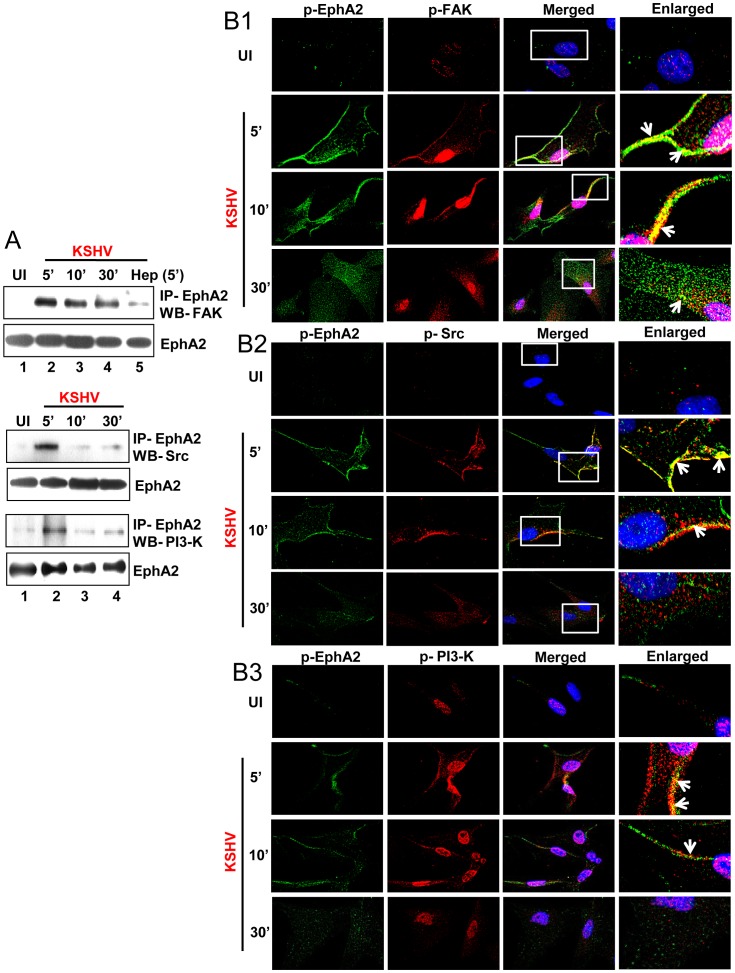
EphA2 recruits KSHV-induced signal molecules necessary for virus entry. (**A**) Serum-starved HFF cells were either mock infected or KSHV infected for the indicated time points and immunoprecipitated with EphA2 antibody followed by Western blot analysis for FAK, Src and PI3-K. For heparin treatment of KSHV, KSHV was pre-incubated with heparin (100 µg/ml) for 1 h at 37°C and used for infecting cells for 5 min followed by cell lysate preparation to confirm specificity of virus induced signaling. The blots were stripped and reprobed for total EphA2 as indicated. (**B**) Serum-starved HFF cells either uninfected or infected with KSHV for the indicated time points were subjected to immunofluorescence assay using rabbit p-EphA2 and either mouse anti-p-FAK (**B1**), anti-p-Src (**B2**) or anti-p-PI3-K (**B3**) antibodies for 2 h at room temperature followed by staining with anti-rabbit Alexa fluor 488 and anti-mouse Alexa fluor 594, respectively. Representative 2D convoluted images are shown. The white boxes within the merged panels are shown as enlarged pictures and the white arrows represent colocalization of the indicated molecules.

Our earlier reports suggest that interaction of KSHV with integrins and other receptors activates the host's pre-existing signal molecules such as FAK, Src and PI3-K which are known to regulate endocytosis [6]. Efficient tyrosine phosphorylation of EphA2 and its association with the activated endocytic effectors c-Cbl, clathrin, clathrin adaptors and myosin IIA as revealed from our data prompted us to elucidate whether EphA2 interacts with these KSHV-induced signal molecules. Mock infected or KSHV-infected HFF cell lysates were IP-ed with anti-EphA2 antibodies followed by Western blotting for FAK, Src and PI3-K. Compared to mock infection, EphA2-immunoprecipitates showed increased association with FAK, Src and PI3-K-p85 as early as 5 min p.i. which decreased by 10 and 30 min p.i. (Figure 6A). The association of EphA2 with FAK was greatly decreased in infection with heparin pretreated KSHV which demonstrated the specificity of virus induced association of these signal molecules with EphA2 (Figure 6A). These were further verified by immunofluorescence analyses which demonstrated the colocalization of p-EphA2 with p-FAK (Figure 6B1), p-Src (Figure 6B2) and p-PI3-K-p85 (Figure 6B3) predominantly at the peripheral region of HFF cells early during KSHV infection (Figures 6 B1, B2 and B3, yellow spots indicated by white arrows). These results strongly suggested that increased levels of pFAK, pSrc and pPI3-K molecules are recruited by activated EphA2 to the KSHV-macromolecular internalization signaling complex critical for endocytosis.

### EphA2 knockdown negatively regulates the activation of endocytic effector molecules and KSHV entry associated signaling

**Figure 7:**
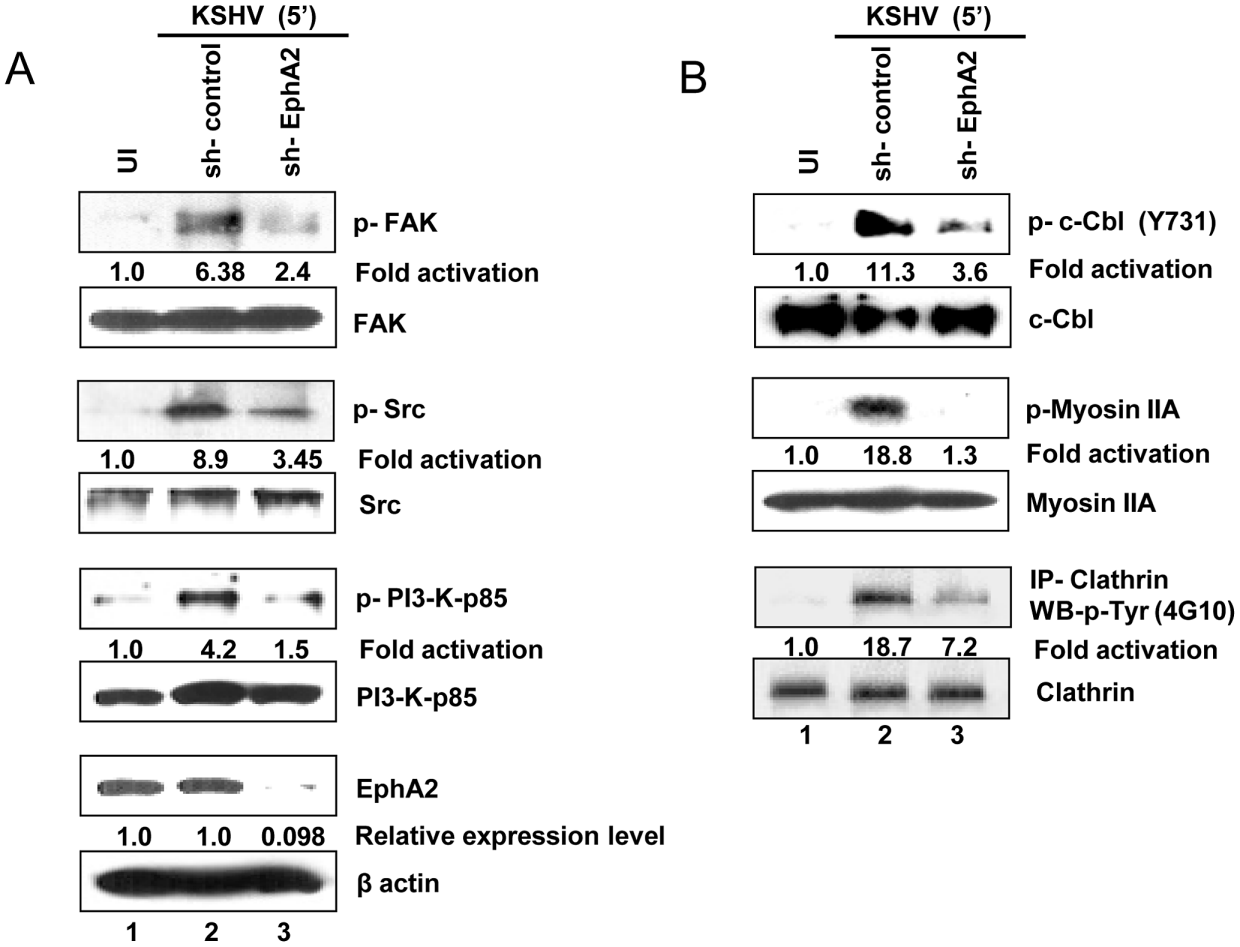
Effect of EphA2 knockdown on the activation of KSHV induced signalling molecules as well as endocytic effectors. (**A**) Control or EphA2 shRNA-transduced HFF cells were either mock infected (UI) or infected with KSHV (30 DNA copies/cell) for the indicated time period and subjected to Western blot analysis for phospho-FAK (p-FAK) (Y397), phospho-Src (Y-416) and phospho-PI3-K (Y458). Blots were stripped and reprobed for the respective total FAK, Src and PI3-K. Efficiency of EphA2 knockdown was analyzed by Western blot with EphA2 antibody and β-actin was used as loading control. (**B**) Control or EphA2 shRNA transduced HFF cells either mock infected or infected with KSHV (30 DNA copies/cell) were subjected to Western blot analysis for activation (phosphorylation) of the indicated endocytic effector molecules (c-Cbl, myosin IIA and clathrin). For clathrin activation, cell lysates were subjected to immunoprecipitation with clathrin heavy chain antibody followed by Western blot analysis with anti-phosphotyrosine antibody (4G10). Blots were reprobed for the respective total c-Cbl, myosin IIA and clathrin for equal expression. The levels of fold activation are indicated.

The role of functional EphA2 in the regulation of KSHV entry associated signaling in relation to efficient virus internalization was ascertained by analyzing the effect of EphA2 knockdown on the activation of effectors such as c-Cbl, myosin IIA and clathrin as well as on selective activation and amplification of FAK, Src and PI3-K-p85 signal molecules. HFF cells transduced with control or EphA2 shRNA were infected with KSHV for 5 min and analyzed by Western blotting for the phosphorylation of the above molecules involved in entry associated events. Compared to control shRNA, ≥90% of EphA2 was knocked down with sh-EphA2 in HFF cells (Figure 7A, seventh panel). While the uninfected cells showed minimal activation, control shRNA cells upon KSHV infection for 5 min showed 6.4, 8.9 and 4.2-fold increase in p-FAK, p-Src and p-PI3-K-p85 levels, respectively (Figure 7A), and 11.3, 18.8 and 18.7-fold increase in phosphorylation of c-Cbl, myosin IIA and clathrin heavy chain, respectively (Figure 7B). In contrast, knocking down EphA2 reduced the activation of FAK, Src and PI3-K by >60% (4.5, 5.5 and 2.7-fold reduction, respectively) (Figure 7A) and also reduced by >75% (7.7 fold), >90% (17.5 fold) and >70% (10.9 fold) in phosphorylation levels of c-Cbl, myosin IIA and clathrin, respectively (Figure 7B). However, there were no marked changes in the respective total expression levels of these molecules which also showed that there were no off target effects by EphA2-shRNA.

KSHV interaction with integrin in the absence of EphA2 could be the potential reason for the observed low levels of FAK, Src and PI3-K phosphorylation in EphA2 knockdown cells. Nevertheless, the observed significant reduction in p-FAK, p-Src, p-PI3-K, p-c-Cbl, p-myosin IIA and p-clathrin due to EphA2 knockdown suggested that EphA2 must be acting as the master coordinator to amplify these signals to regulate virus entry, and reduced levels of these signals could be the potential reason for the dramatic reduction in KSHV entry and infection. These studies clearly demonstrated the importance of functional EphA2 in the modulation of signal amplifications critical for c-Cbl-myosin- dependent clathrin mediated endocytosis of KSHV in HFF cells.

### EphA2 knockdown impairs KSHV trafficking to early endosomes due to a defect in clathrin mediated entry

**Figure 8:**
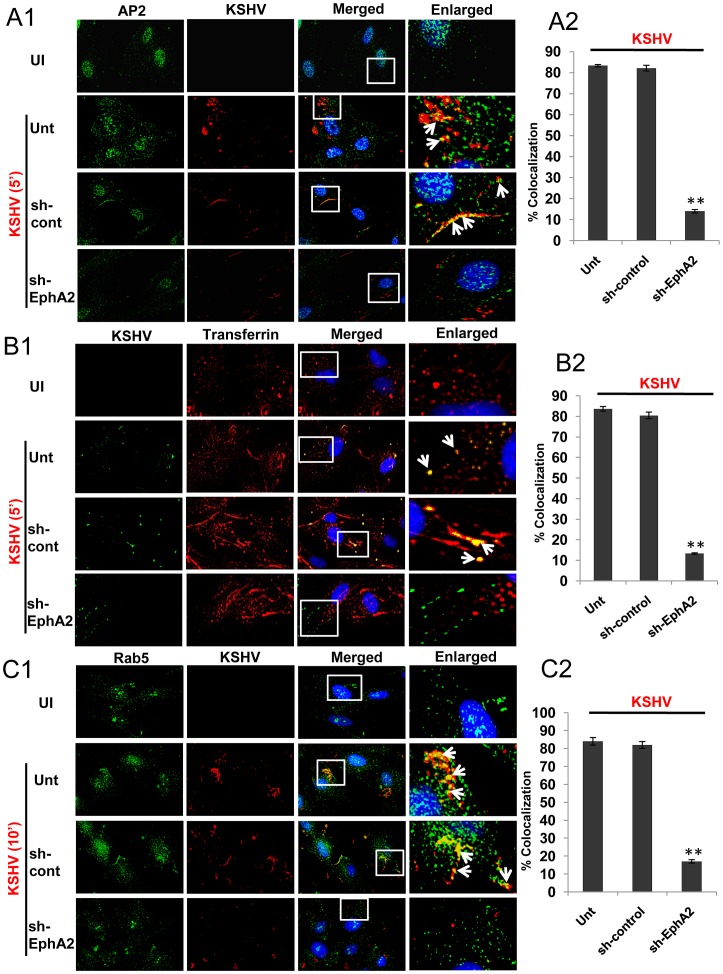
EphA2 knockdown has a negative effect on KSHV trafficking in the early endosome due to defective endocytosis. (**A**) Control sh-RNA or EphA2-sh-RNA-transduced or untransduced HFF cells were serum starved and either mock infected (UI) or infected with KSHV (30 DNA copies/cell) for 5 min at 37°C and were processed for immunofluorescence assay using clathrin adaptor AP2 and mouse-gpK8.1A antibodies for 2 h at room temperature followed by staining with anti-rabbit Alexa fluor 488 or mouse Alexa fluor 594. The enlarged pictures represent boxed regions within the merged panels and the white arrows represent colocalization of the indicated molecules. Representative 2D convoluted images are shown (**A1**). Quantification of KSHV particles colocalized with AP2 molecules was performed by counting three different fields having atleast 10 cells each and error bars represent mean ± SD, ** represents p<0.01 (**A2**). (**B**) Control or EphA2 sh-RNA-transduced HFF cells either mock infected (UI) or infected with medium containing Alexa 594-transferrin along with KSHV (30 DNA copies/cell) for 5 min at 37°C were subjected to immunofluorescence assay using rabbit anti-gB antibody for 2 h at room temperature followed by staining with anti-rabbit Alexa fluor 488. Representative 2D convoluted images are shown (**B1**). The enlarged pictures represent boxed regions within the merged panels and the white arrows represent colocalization of the indicated molecules. Quantification of KSHV particles colocalized with Transferrin molecules was performed by counting three fields with atleast 10 cells each and error bars represent mean ± SD, ** represents p<0.01 (**B2**). (**C**) (**C1**) Serum starved control sh-RNA or EphA2 sh-RNA transduced HFF cells, either mock infected or infected with KSHV for 10 min at 37°C, were washed and processed for immunofluorescence using rabbit anti Rab5 and mouse anti-gpK8.1A antibodies for 2 h at room temperature followed by staining with anti-rabbit Alexa fluor 488 or mouse Alexa fluor 594. Representative 2D convoluted images are shown. The white boxes within the merged panels are shown as enlarged pictures and the white arrows represent colocalization of the indicated molecules. (**C2**) Quantitation of KSHV accumulated in Rab5 positive endosomes was performed by counting three different fields having at least 10 cells each and error bars represent mean ± SD, ** represents p<0.01.

Since KSHV-induced signaling is highly coupled to its endocytosis [6] and as we observed EphA2 dependent activation and association of endocytic effectors (c-Cbl, myosin IIA and clathrin) and regulation of KSHV-induced signaling, we further characterized the role of EphA2 in endocytic trafficking of KSHV via a clathrin dependent route. We tracked the internalization of KSHV in EphA2 knockdown HFF cells with respect to control shRNA transduced cells. Within 5 min p.i., a double labeled immunofluorescence assay showed substantial colocalization of KSHV particles with clathrin adaptor AP2 in control shRNA transduced cells (Figure 8A1, third panel, white arrows) with ≥80% colocalization frequency (Figure 8A2) which is similar to virus only infected positive controls (Figure 8A, second panel, Unt; white arrows). However, in shEphA2 transduced cells, such colocalization was not significantly present (Figure 8A1, bottom panel) (<15% colocalization frequency, p<0.01, Figure 8A2) suggesting that initiation of clathrin mediated endocytosis of KSHV is mostly dependent on EphA2.

A similar colocalization was also observed within 5 min p.i. between KSHV particles and Alexa 594-conjugated transferrin, a physiological ligand known to enter cells specifically via clathrin mediated endocytosis, in control shRNA transduced or untransduced cells infected with KSHV (Figure 8B1,Unt; white arrows) (≥80% colocalization frequency, Figure 8B2) which was less evident in virus infected EphA2-shRNA transduced cells (Figure 8B1, bottom panel) (<15% colocalization frequency, p<0.01, Figure 8B2). This result suggested that KSHV was internalized with clathrin dependent endocytic marker transferrin and further validated the involvement of EphA2 in clathrin mediated entry of KSHV in HFF cells.

We next investigated the role of EphA2 in KSHV trafficking by tracking its uptake into Rab5 positive endosomes in control and EphA2 shRNA-transduced HFF cells. In control shRNA transduced cells KSHV localized predominantly in Rab5 positive endosomes which is similar to untransduced virus infected positive control cells (Figure 8 C1, Unt; white arrows) with >80% colocalization frequency (Figure 8C2). In contrast, cells transduced with EphA2 shRNA showed significantly reduced (<20%) colocalization (p<0.01) of virus with Rab5 (Figures 8 C1 and C2). Taken together, these studies demonstrated that EphA2 clearly has a positive role in clathrin mediated entry of KSHV and its trafficking towards early endosomes presumably for productive infection.

### EphA2 undergoes polyubiquitination mediated by c-Cbl early during KSHV infection

In endothelial cells, KSHV induced the association of EphA2 with c-Cbl, a critical endocytic effector molecule with a well characterized E3-ubiquitin ligase activity [28]. It is also well documented that many receptors, including receptor tyrosine kinases, undergo ligand-dependent ubiquitination mediated by c-Cbl [29], [30]. Receptor ubiquitination has been recognized as an internalization signal. Moreover, the fate of internalized receptors also varies with the pattern of ubiquitination [31], [32]. Furthermore, our studies already showed that EphA2 interacts with the adaptor molecule Epsin15 (Figure 3) which is known to mediate the interaction between ubiquitinated cargo molecules and clathrin, an inducer of internalization [33], [34]. Therefore, we theorized that early during KSHV infection of HFF cells, EphA2 may undergo c-Cbl mediated ubiquitination to assist in clathrin-dependent virus internalization.

**Figure 9:**
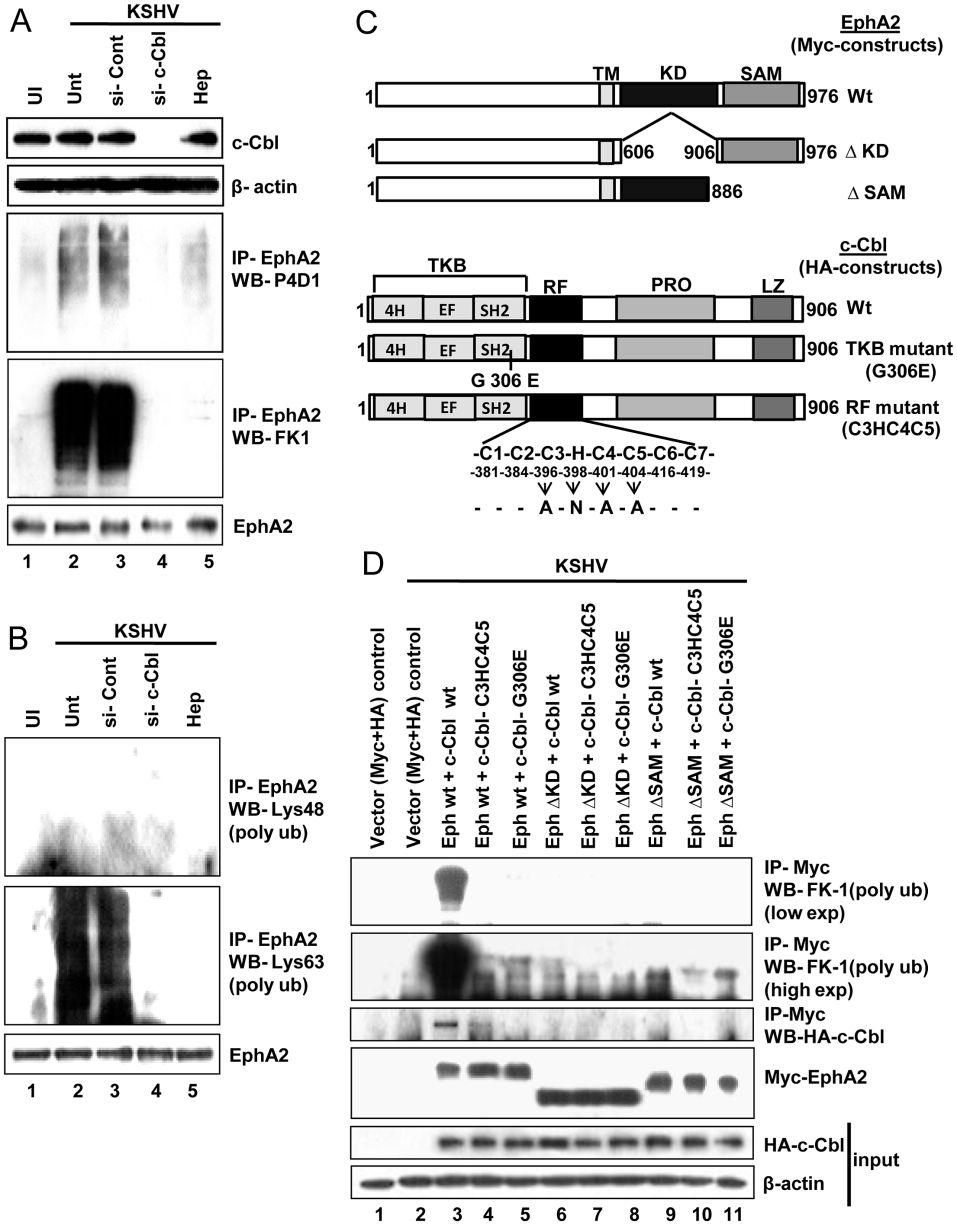
c-Cbl directed polyubiquitination of EphA2 early during KSHV infection. (**A**) Control or c-Cbl-siRNA-transfected HFF cells either mock infected (UN) or infected with KSHV (30 DNA copies/cell) for 5 min were lysed and subjected to Western blot analysis for c-Cbl expression (First panel). β-actin was used as loading control (second panel). 200 µg of the lysates prepared under the stringent conditions for ubiquitination were immunoprecipitated with anti-EphA2 antibody followed by Western blotting with either total anti-ubiquitin antibody P4D1 (mouse anti-mono- and anti-polyubiquitin antibody) (third panel) or FK-1 antibody detecting polyubiquitination (Fourth panel). The blot was stripped and reprobed for EphA2 (Fifth panel). For heparin treatment of KSHV, KSHV was pre-incubated with heparin (100 µg/ml) for 1 h at 37°C and used for infecting cells for 5 min. (**B**) Validation of the differential nature of EphA2 polyubiquitination. 200 µg of the whole-cell lysate prepared under stringent conditions from fig. 9. A experiment was immunoprecipitated with rabbit anti-EphA2 antibody and was subjected to Western blot analysis for either Lys-48 (first panel) or Lys-63 (second panel) specific polyubiquitination. Blots were stripped and reprobed for EphA2. For heparin treatment of KSHV, KSHV was pre-incubated with heparin (100 µg/ml) for 1 h at 37°C and used for infecting cells for 5 min followed by cell lysate preparation to confirm specificity of virus induced events. (**C**) Schematic representation of Myc-EphA2 deletion mutants and HA-c-Cbl substitution mutants in comparison to the wild type proteins (Wt). For EphA2 constructs, numbers indicate amino acid position within the sequence. TM, Transmembrane; KD, Kinase domain; SAM, Sterile alpha motif. For c-Cbl mutants amino acid substitutions are indicated. Glycine (G) was substituted to Glutamic acid (E). C1–C7 represents conserved Cysteine residues and H represents the Histidine. Substitutions to either Alanine (A) or Asparagine (N) were made at the indicated positions. TKB, Tyrosine kinase binding domain; RF, Ring finger domain; PRO, Proline rich region; LZ, Leucine zipper. (**D**) HEK 293 cells were transiently cotransfected with Myc-EphA2 (Wt, ΔKD and ΔSAM) and HA-c-Cbl (Wt, Cbl-C3HC4C5 and Cbl-G306E) as indicated or with vector controls. 48 h post-transfection, cells were mock infected or infected with KSHV (30 DNA copies/cell) for 5 min and cell lysates prepared under stringent conditions were used for coimmunoprecipitation with Myc antibody followed by Western blot analysis with polyubiquitin specific FK-1 (First panel, low exposure blot; second panel, high exposure blot). To analyse Myc-EphA2 and HA-cCbl interaction in a separate experiment, lysates from HEK 293 cells overexpressing different combinations of Myc-and HA-tagged Wt and mutant proteins were immunoprecipitated with anti-Myc antibody followed by western blot with HA antibody (Third panel). Blots were stripped and reprobed for Myc (Fourth panel). Whole cell lysates were subjected to Western blot analysis for HA expression (input) (Fifth panel) and β-actin from whole cell lysates was used as loading control (Sixth panel).

To test this hypothesis, we downregulated the expression of c-Cbl in HFF cells by si-RNA and observed >90% inhibition in c-Cbl expression relative to controls (Figure 9A, upper two panels). Next we checked the ubiquitination of EphA2 after immunoprecipitation of the uninfected, control or si-c-Cbl treated KSHV infected HFF whole cell lysates with EphA2 antibody followed by Western blotting with P4D1 monoclonal antibody known to efficiently recognize both mono- and polyubiquitin [35]. We also used FK-1 monoclonal antibody which specifically detects polyubiquitin but not monoubiquitin [35]. Cell lysates prepared under very stringent conditions as described in the materials and methods section were used in these experiments to detect ubiquitination of proteins. Compared to uninfected cells, as early as 5 min p.i. in infected cells, increased ubiquitination of EphA2s was observed with P4D1 antibody in control si-RNA treated cells, which was similar to cells infected with KSHV only (Figure 9A, third panel, lanes 2 and 3). In contrast, very poor or negligible ubiquitination was detected in si-c-Cbl treated HFF cells (Figure 9A, third panel, lane 4). When experiments were conducted to discriminate the nature of ubiquitination, a similar result was observed with polyubiquitin specific FK-1 antibody where an intense smeary pattern resembling polyubiquitination was seen in control si-RNA treated KSHV infected cells similar to virus only infection (Unt), whereas negligible or no ubiquitination was observed in si-c-Cbl treated cells (Figure 9A, fourth panel, lanes 2 and 3 vs. lane 4). This result clearly demonstrated the involvement of c-Cbl promoting polyubiquitination of EphA2 early during KSHV infection.

To prove that EphA2 polyubiquitination is a result of KSHV infection, we used heparin-treated KSHV for infection. As expected, EphA2 ubiquitination was significantly reduced by heparin treatment of KSHV (Figure 9A, third and fourth panels, lane 5), suggesting that KSHV binding in infected cells triggers polyubiquitination of the EphA2 receptor.

### EphA2 undergoes c-Cbl mediated Lys63 linked polyubiquitination early during KSHV infection

The typical polyubiquitin chains reported include either K48-linked chains which have been studied in the context of protein degradation [36] or K63-linked chains which have been implicated in a variety of non-proteolytic functions including mediators of novel signaling [37]. Therefore, we examined the composition of the EphA2 polyubiquitin chains in a similar experiment as before by probing with monoclonal antibodies specific for either K48–linked or K63-linked polyubiquitin chains. As shown in Figure 9B second panel, the Lys63 linkage but not the Lys48 linkage (Figure 9B, first panel) was most strongly detected in control siRNA treated cells similar to KSHV infection alone (5 min p.i.;Unt) but negligible or very low levels in cells treated with c-Cbl siRNA. We also observed faint lys 48-linked polyubiquitination of EphA2 (Figure 9 B, first panel) which could be due to autouiquitination of c-Cbl and/or due to the induction of EphA2 by its ephA1 ligand. Nevertheless, these results clearly suggested Lys63-linked polyubiquitination of EphA2 directed by c-Cbl early during KSHV infection.

### EphA2 polyubiquitination is facilitated by association with E3-ligase c-Cbl early during KSHV infection

In search of the regions responsible for physical association between EphA2 and c-Cbl that have functional relevance with EphA2 ubiquitination we used specific deletion mutants of Myc-tagged EphA2 namely EphA2-Kinase Dead (Eph-ΔKD) and EphA2-sterile alpha motif (Eph-ΔSAM) mutants, and HA-tagged c-Cbl mutants such as c-Cbl RING Finger (RF) domain mutant (c-Cbl-C3HC4C5) and c-Cbl Tyrosine Kinase Binding (TKB) mutant (c-Cbl-G305E) along with their respective Wild-type (Wt) constructs (Figure 9C). Since HFF cells are not easily transfectable, we used 293 cells for this study. Each of the Myc-tagged EphA2 constructs was cotransfected with each of the HA-tagged c-Cbl constructs and the cell lysates from the KSHV infected or uninfected cells were used for ubiquitination analysis of Myc-EphA2. Cell lysates prepared under stringent conditions were immunoprecipitated using anti-Myc antibody followed by immunoblotting with polyubiquitin specific FK1 antibody (Fig. 9D, first and second panels). To delineate the interaction between Myc-EphA2 and HA-cCbl, lysates from HEK293 cells overexpressing different combinations of Myc-and HA-tagged Wt and mutant proteins were subjected to coimmunoprecipitation with anti-Myc antibody followed by western blot with anti-HA antibody ((Fig. 9D, third panel). Expression of Wt and deletion mutant EphA2 proteins was verified by immunoblotting with Myc antibody of the same blot (Figure 9D, panel 4), and expression of Wt and mutant c-Cbl proteins was verified by immunoblotting the cell lysates with anti-HA antibody (Figure 9D, panel 5).

Strong polyubiquitination was detected only in Wt-EphA2-Myc precipitated with Wt-HA-c-Cbl (Figure 9D, second panel, lane 3). For clarity, a low exposure of the same blot is shown in which we observed the smeared bands of Myc-tagged EphA2 (Figure 9D, first panel, lane 3). In contrast, negligible or no polyubiquitination was observed in each of the other EphA2 precipitates (Figure 9D, first and second panels, lanes 4–11) compared to the negative control (Myc-+HA- vectors only) (Figure 9D, first and second panels, lanes 1–2). Interestingly, only Wt-c-Cbl appeared to be coimmunoprecipitated with Wt-EphA2 (Figure 9D, third panel, lane 3) but very little or no c-Cbl, whether Wt or c-Cbl-C3HC4C5 or c-Cbl-G306E, was observed to be coimmunoprecipitated with EphA2-ΔKD or EphA2-ΔSAM (Figure 9D, third panel, lanes 6–11). Similarly, very poor or no association of Wt-EphA2 was detected with c-Cbl-C3HC4C5 or c-Cbl-G306E (Figure 9D, third panel, lanes 4–5). The intense polyubiquitinated Myc-EphA2 bands observed in Figure 9D, first and second panel lane 3 could be due to strong interaction between overexpressed EphA2 and c-Cbl upon KSHV infection. This is similar to the intense smeary bands of polyubiquitinated bands of EphA2 seen in Fig. 9 A and B.

These results demonstrated that physical association of EphA2 with c-Cbl occurs probably with the aid of both the EphA2 tyrosine kinase (TK) and SAM domains and facilitated by the presence c-Cbl RF and TKB domains. Other than the Wt-EphA2 and c-Cbl cotransfection, the absence of polyubiquitination in KSHV infected cells corresponding to cotransfection with each of the other Myc-EphA2 and HA-c-Cbl mutants clearly suggested the functional significance of c-Cbl mediated KSHV induced polyubiquitination of EphA2 involving association of c-Cbl –TKB and –RF domain along with EphA2 TK and SAM domains.

### c-Cbl knockdown disrupts the association of EphA2 with clathrin early during KSHV infection in HFF cells

**Figure 10:**
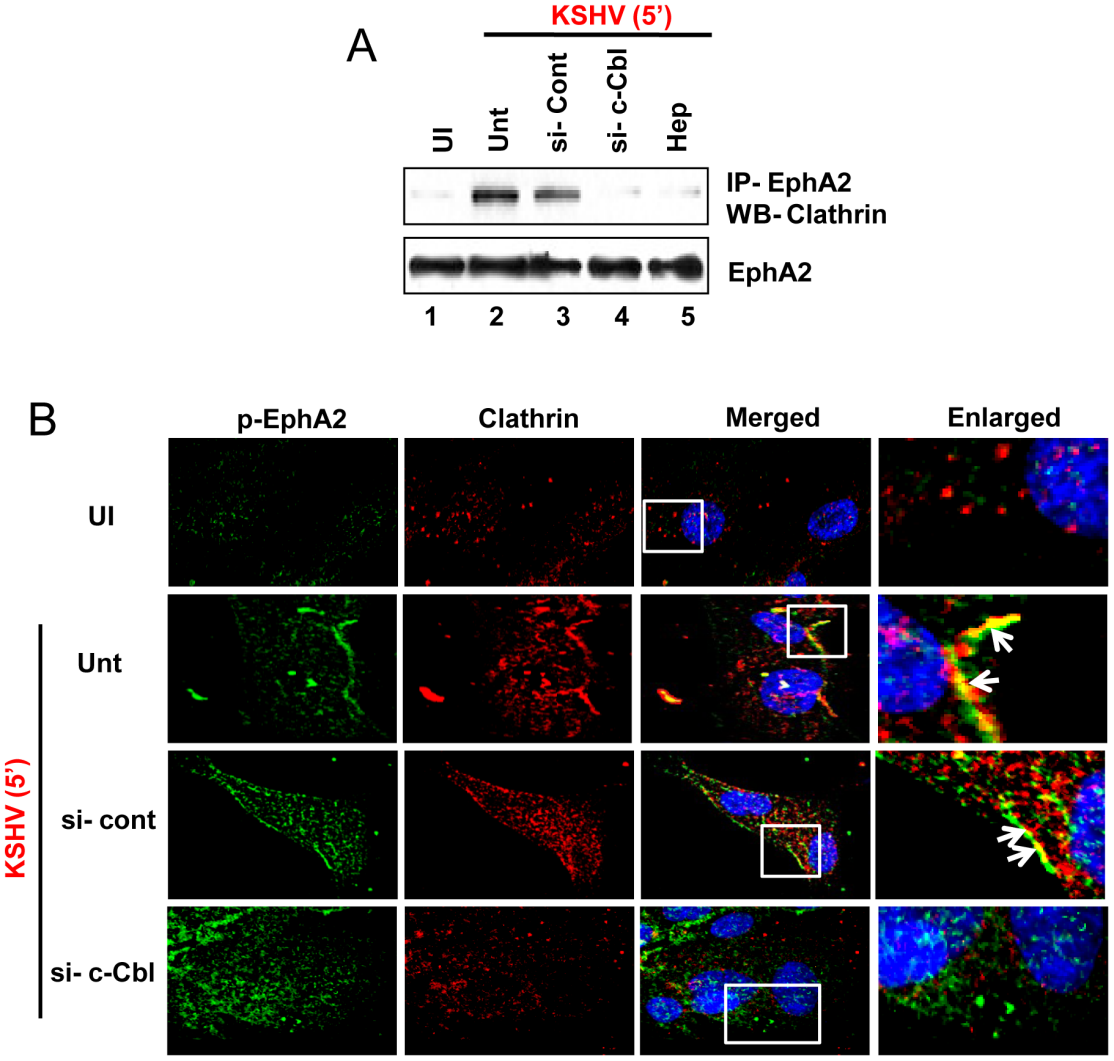
Effect of c-Cbl knockdown on the association of EphA2 with clathrin. (**A**) Control or c-Cbl-si-RNA-transfected HFF cells either mock infected (UI) or infected with KSHV (30 DNA copies/cell) or KSHV pre-incubated with heparin (100 µg/ml) for 1 h at 37°C, for 5 min were lysed and subjected to immunoprecipitation with EphA2 antibody followed by western blot analysis with anti-Clathrin antibody. Blot was stripped and reprobed for EphA2. (**B**) Serum-starved control si-RNA or c-Cbl-si-RNA transfected HFF cells either uninfected or infected with KSHV for 5 min were subjected to immunofluorescence assay using rabbit p-EphA2 and mouse anti-clathrin antibodies overnight at 4°C followed by staining with anti-rabbit Alexa fluor 488 and anti-mouse Alexa fluor 594, respectively. Representative 2D convoluted images are shown. The white boxes within the merged panels are shown as enlarged pictures and the white arrows represent colocalization of the indicated molecules.

The fact that receptor ubiquitination serves as a better internalization signal for clathrin adaptors during clathrin mediated endocytosis [38], [39], [40] and c-Cbl directed polyubiquitination of EphA2 in our results prompted us to investigate the functional implications of this event in clathrin mediated endocytosis. Therefore, we verified the consequence of c-Cbl knockdown affecting the association of EphA2 with clathrin during KSHV infection of HFF cells. Upon KSHV infection (5 min), when cell lysates of HFF cells pretreated with control or c-Cbl siRNA were immunoprecipitated using anti-EphA2 antibody, strong co-IP of EphA2 and clathrin heavy chain was observed in control siRNA treated cells similar to virus only infected cells (Figure 10A, upper panel, lanes 2 and 3). However, such co-IP was significantly reduced (∼8 fold) in cells treated with c-Cbl siRNA (Figure 10A, upper panel, lane 4). Consistent with this result, in immunofluorescence studies, knockdown of c-Cbl showed no appreciable colocalization between p-EphA2 and clathrin as evident from the non-coherent pattern of green (p-EphA2) and red (clathrin) signals (Figure 10B, panel 4). In contrast, substantial colocalization of EphA2 with clathrin was observed in cells treated with control siRNA similar to virus only infection (Figure 10B, panels 2 (Unt) and 3 (si-cont), white arrows). Altogether, these results clearly demonstrated the active participation of c-Cbl in regulating KSHV induced association of EphA2 with clathrin and thus influencing clathrin mediated endocytosis.

### c-Cbl ‘loss of function’ impairs association of KSHV with clathrin but not with EphA2 and directs the minimally entered viral particles into the lysosome

**Figure 11:**
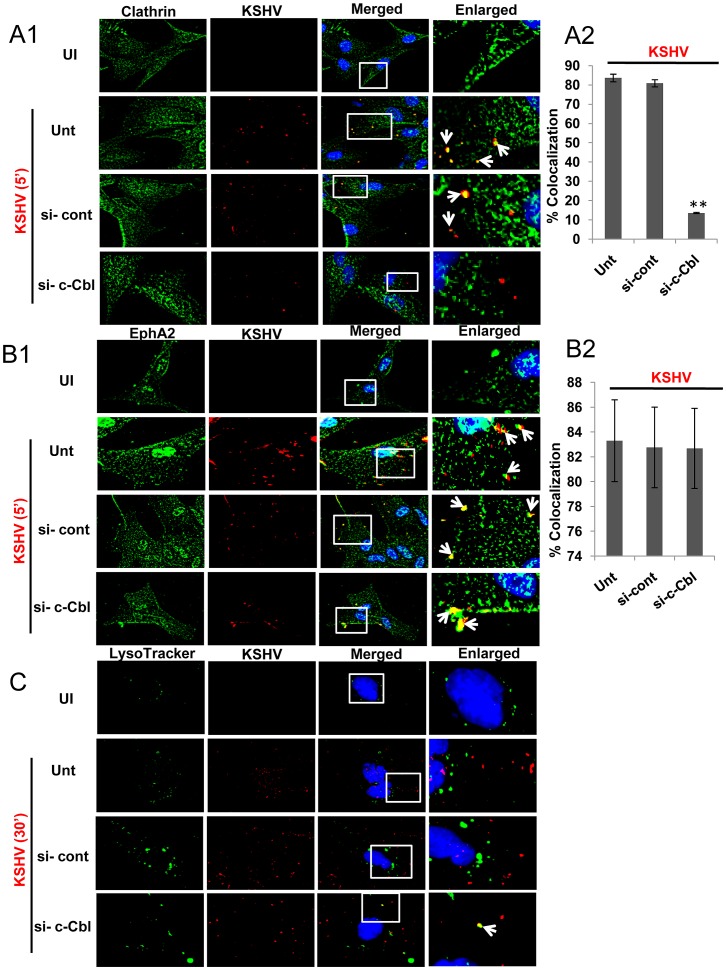
c-Cbl regulates clathrin mediated productive entry of KSHV without affecting its association with EphA2. (**A and B**) Serum starved, control si-RNA or c-Cbl-si-RNA transfected, HFF cells either uninfected or infected with KSHV for 5 min were subjected to immunofluorescence assay using anti-rabbit KSHV gB and mouse anti-clathrin antibodies (**A1**) or anti-rabbit EphA2 and anti-mouse KSHV gpK8.1 antibodies (B1), respectively, overnight at 4°C followed by staining with either anti-rabbit Alexa 594 and anti-mouse Alexa 488 (**A1**) or vice versa (**B1**). Representative 2D convoluted images are shown. The enlarged pictures represent boxed regions within the merged panels and the white arrows represent colocalization of the indicated molecules. (**A2 and B2**) Quantification of KSHV colocalized with clathrin or EphA2 was performed by counting three different fields having atleast 10 cells each and error bars represent mean ± SD, ** represents p<0.01. (**C**) Control or c-Cbl-si-RNA-transfected HFF cells were incubated with medium containing LysoTracker green along with or without KSHV (30 DNA copies/cell) for 30 min at 37°C and were processed for immunofluorescence assay using anti-gB antibody followed by Alexa 594 anti-rabbit secondary antibody. Representative 2D convoluted images are shown. The white boxes within the merged panels are shown as enlarged pictures and the white arrows represent colocalization of the indicated molecules.

We rationalized that if EphA2 that is critically associated with KSHV entry undergoes c-Cbl directed polyubiquitination, and c-Cbl knockdown disrupts association between EphA2 and clathrin, then c-Cbl ‘loss of function’ should also impair clathrin mediated endocytosis of KSHV in HFF cells. Hence, we sought to characterize the functional effect of c-Cbl on the association of KSHV with clathrin upon c-Cbl knockdown in HFF cells. KSHV colocalized appreciably with clathrin by 5 min. p.i with some viruses located inside in control siRNA treated cells similar to virus only infected cells (Figure 11A1, second (Unt) and third (si-cont) panels) (≥80% colocalization frequency, Figure 11A2). In contrast, no significant colocalization between KSHV and clathrin was observed on c-Cbl siRNA treatment as suggested from the unsynchronized green and red signals (Figure 11A1, fourth panel) (<15% colocalization frequency, Fiure 11A2); moreover, the majority of the viruses were present at the outer cell periphery (Figure 11A1, fourth panel). However, we still observed significant colocalization between EphA2 with KSHV (Figure 11B1) even in c-Cbl knockdown HFF cells, similar to control siRNA treatment or virus only infection in the absence of any si-RNA as validated from synchronous green (EphA2) and red (KSHV) signal (Figure 11B1, second, third and fourth panel) (∼80% colocalization frequency, Figure 11B2). These results demonstrated the potential involvement of c-Cbl in regulating clathrin mediated KSHV entry into HFF cells without disturbing the association of KSHV with EphA2, one of its receptors, and suggested that interaction of virus with EphA2 is followed by subsequent c-Cbl directed clathrin dependent entry.

Given the fact that c-Cbl knockdown impaired (i) EphA2 polyubiquitination (K63 type), (ii) association of EphA2 with clathrin, (iii) association of KSHV with clathrin without any disturbance of prior interaction of KSHV with EphA2 and the observation that EphA2 knockdown resulted in ∼80% KSHV entry inhibition but >90% inhibition of viral gene expression, we speculated whether c-Cbl directed EphA2 polyubiquitination may be registered as an internalization signal for clathrin mediated virus entry into HFF cells. Since we observed ∼15–20% entry of input virus in the presence of EphA2sh-RNA, it prompted us to investigate the fate of the entered viral particles in the context of c-Cbl knockdown. We tracked the internalized KSHV particles, by 30 min. p.i., in control and si-c-CblRNA treated HFF cells with LysoTracker, a basophilic lysosomal marker. While in si-control or virus only infected cells, KSHV (red) did not colocalize with LysoTracker (green) (Figure 11C, second and third panels), in c-Cbl knockdown cells, the majority of the virus particles remained at the cell periphery and the very minimal amount of internalized virus was found to colocalize with LysoTracker. This result suggested entry and trafficking of virus towards the lysosome and consequently a nonproductive infection possibly through a non-c-Cbl mediated mechanism. Taken together, our results clearly demonstrated that c-Cbl dependent EphA2 polyubiquitination is essential for clathrin mediated entry of KSHV in HFF cells which results in a productive infection leading to establishment of latency.

## Discussion

**Figure 12:**
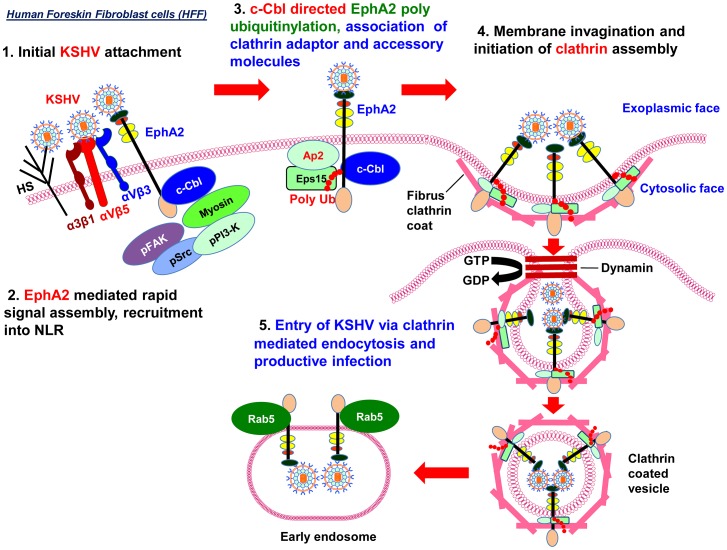
Schematic model depicting the proposed mechanism of EphA2 dependent KSHV entry in human foreskin fibroblast cells via clathrin mediated endocytosis. (**1**) Binding and interaction of KSHV with HFF cell surface heparan sulfate and integrins (αVβ3, αVβ5 or α3β1) is followed by their association with EphA2 in the non-LR region. (**2**) EphA2 coordinates formation of active signaling complex among integrins, c-Cbl and myosin IIA with concomitant induction of FAK, Src and PI3-K signaling necessary for endocytic entry of associated virus. (**3**) c-Cbl directed polyubiquitination (K63-linked) of EphA2 helps interaction with accessory proteins Epsin15 and adaptor protein AP-2. (**4**) These interactions promote activation, recruitment and assembly of clathrin to the formation of clathrin coated pits (CCP). (**5**) Together, such complex signaling and associated events trigger internalization of KSHV into clathrin coated pits, dynamin dependent release of endocytic vesicles [22] that lead to trafficking of KSHV into the Rab5 early endosome followed by a productive KSHV infection and gene expression.

Our comprehensive studies presented here demonstrate that EphA2 plays a crucial role in coordinating and amplifying KSHV induced signaling essential for virus internalization through clathrin mediated endocytosis (CME) in human fibroblast cells (Figure 12). In the course of studies conducted to delineate the mechanism underlying EphA2 dependent modulation clearly demonstrated for the first time the following findings: i) EphA2 associates with and influences the tyrosine phosphorylation of clathrin heavy chain; ii) EphA2 plays a role in clathrin mediated endocytosis of a virus; iii) c-Cbl E3 ubiquitin ligase actively participates in KSHV entry by polyubiquitinating EphA2 which is necessary for EphA2 to be effective as an internalization signal for CME of KSHV and trafficking towards the endosome but not lysosome for successful infection. For clarity and better understanding, we have summarized in the following sections the potential implications of the multiple roles of EphA2 and c-Cbl in KSHV entry.

### Role of EphA2 in KSHV entry into HFF cells

The Eph family of receptor tyrosine kinases (RTK) and their membrane bound ligands, known as Ephrins, exert bidirectional signaling where signaling through ligands is “reverse signaling” and through Eph receptors represents “forward signaling” [41]. They are known to mediate diverse activities such as integrin-associated signaling, effects on the actin cytoskeleton, cell-substrate adhesion, intracellular junctions, cell shape and cell movement [41], [42], [43], [44], with broad implications in neovascularization and oncogenesis [45], [46]. Ephrin receptors have also been shown to be the center of signaling crosstalk between integrins, PI3-K, and Rho-GTPases [20], [47], which are also induced early during KSHV infection [6]. Our current study shows that EphA2 is predominantly a non-LR partitioned molecule in HFF cells which is activated by KSHV infection in association with KSHV entry receptors (integrins α3β1, αVβ3, αVβ5) at the cell periphery. Very poor or absence of integrin association with EphA2 in uninfected cells signifies this to be a virus induced event. This finding is consistent with the previous reports where Ephrin-EphR has been shown to be involved in regulating an assortment of integrin signaling pathways [42], [43], [47], [48].

Our studies demonstrating the activation of c-Cbl, myosin IIA along with clathrin, substantial colocalization of c-Cbl and myosin and the association of EphA2 with c-Cbl and clathrin during KSHV infection particularly at early time points (5 min and 10 min p.i.) indicate an active macromolecular complex comprised of integrin-c-Cbl-myosin-EphA2, critical for clathrin mediated internalization of KSHV. The formation of clathrin-coated pits (CCP) at the plasma membrane during the process of receptor-mediated endocytosis requires the interaction of clathrin with adaptors which serve important roles in the regulatory and catalytic mechanism at specific stages in clathrin mediated endocytosis [49]. Clathrin is recruited to CCP by AP2 [50], which has two large subunits involved in the recruitment of accessory proteins [51]. One such protein is Epsin15, which is required for CCP assembly and invagination during CME [52], [53]. Colocalization of activated EphA2 with clathrin (Figure 4B) and association of EphA2 with AP2 and Epsin15 (Figure 3D) very early during KSHV infection further provides strong evidence that the initial internalization of KSHV may proceed via EphA2 dependent clathrin mediated endocytosis. This observation is consistent with the previous finding where ligand induced EphA2 has been implicated in endocytosis and receptor clustering [54], however, involvement of EphA2 in clathrin mediated endocytosis of a virus was not reported before. Predominant activation of clathrin and its association with EphA2 very early during virus infection with concomitant decrease overtime indicates that the process is rapid and occurs at the very initial stage of infection. Since the non-LR region constitutes most of the cellular area enriched with all of the KSHV entry receptors, distribution of EphA2 at the non-LR region of HFF cells probably provides a good platform for the initiation of infection.

### Role of EphA2-host signal molecules in KSHV entry into HFF cells

Selective modulation of cellular signaling is crucial for endocytosis regulation [55]. However, there is little information regarding the molecules involved in signal assembly and regulation of virus endocytosis. We observed significant association of EphA2 with FAK, Src and PI3-K as early as 5 min post KSHV infection in HFF cells which decreased from 10 min and 30 min p.i. indicating EphA2 mediated recruitment of these signal molecules. EphA2 knockdown significantly reduced not only the activation of FAK, Src and PI3-K signal molecules but also the endocytic adaptors c-Cbl, myosin IIA and clathrin. Though tyrosine phosphorylation of clathrin heavy chain was implicated in the internalization of bacteria [56], our studies report for the first time that EphA2 influences the tyrosine phosphorylation of clathrin heavy chain during KSHV infection. EphA2 mediated regulation of these molecules along with clathrin further suggests that EphA2 is a master regulator of signal molecules during KSHV entry. Since Src has been shown to be involved in the activation of clathrin and AP2 during clathrin mediated endocytosis [57], the role of EphA2 in the amplification of KSHV-induced Src activation early during KSHV infection suggests that this amplification is essential for clathrin/AP2 activation and formation of CCP.

As shown by our previous studies and in the present study, although KSHV induces the same key molecules during entry of HMVEC-d and HFF cells, entry into fibroblast cells and into endothelial cells involves two different endocytic pathways. This is potentially due to where EphA2 is distributed in these cells. EphA2 is detected strictly in the LRs of HMVEC-d cells [19]. However, in HFF, EphA2 is distributed mostly in the nonLR region. Our previous studies show that EphA2 interaction is vital for virus entry in HMVEC-d cells and KSHV induced c-Cbl promotes the rapid virus bound integrin receptors translocation into LRs of infected cells where KSHV interaction with EphA2 amplifies and couples integrin associated Src and PI-3K signaling with macropinocytosis [19]. In contrast, in our current study since EphA2 is predominantly distributed in the non-LR region of HFF cells, there is probably no need for receptor translocation and KSHV induced interaction of integrin with EphA2 enhances the FAK, Src, PI-3K, c-Cbl and clathrin activation in nonLR region followed by clathrin mediated endocytosis (Fig. 12).

### Role of c-Cbl mediated ubiquitination in KSHV entry in HFF cells

Ubiquitin (Ub) modification of membrane proteins appears to be one of the preferred sorting signals for clathrin mediated internalization where Ub represents a binding surface to clathrin effectors like Epsin or Epsin15 [33], [58]. However, the mechanism of how ubiquitination of viral particles bound to cell surface receptors affects productive viral entry and infection remains to be clarified. c-Cbl E3 ubiquitin ligase not only plays important roles in signal transduction as negative regulators of receptor-based signaling by promoting ubiquitination but also acts as a part of a complex signaling interactome controlling a variety of functions, including internalization and endosomal sorting [30], [59] and as an endocytic modulator during KSHV entry in endothelial cells [18].

In HFF cells, KSHV infection induces the association of EphA2 with c-Cbl very early during infection which directs the K63 specific rather than K48 type of polyubiquitination of EphA2. This is relevant in the context of signaling and endocytosis as K63 linked polyubiquitination has been mostly implicated in a variety of non-proteolytic functions including the endocytosis pathway [37], [60] whereas K48-linked chains have been implicated in protein degradation [36]. Many of the proteins that undergo Ub-dependent internalization are modified by K63-polyUb and some of the key cargo-modifying Ub ligases favor the formation of K63-linked chains probably because a single Ub appended to a model protein may serve as a rather poor internalization signal. For example, TrkA (tropomyosin receptor kinase A, the nerve growth factor receptor), is modified by TRAF6 (tumor necrosis factor receptor-associated factor), an enzyme that favors formation of K63 chains [61]; MHC-I, is modified by KSHV encoded K3-MARCH (membrane-associated RING-CH) ligase and the Ub conjugating enzymes UbcH5/Ubc13, which favor K63-linked chains [62].

Importantly, perturbing the Ub system leading into the inhibition of the formation of K63 chains diminishes internalization. For instance, over expression of mutant UbK63R, which interferes with the formation of K63-polyUb, attenuates internalization of the prolactin receptor, TrkA, and MHC-I [61], [62]. Therefore, the more Ub a cargo carries, the more effective its internalization–most probably because this promotes the interaction of cargo with Ub-sorting receptors, which typically have poor affinity for a single Ub. Formation of a polyUb chain may simply be the simplest mechanism for achieving good binding, especially when a limited number of lysine residues are available on relevant cargo. These events could also be effective in our study and c-Cbl could preferentially participate in K-63 liked polyubiquitination of EphA2 for efficient sorting by Ub-sorting proteins like Epsin 15 associated in clathrin mediated endocytosis of KSHV similar to EGFR endocytosis [63].

Non-association between EphA2-ΔKD and c-Cbl-TKB (c-Cbl-G306E) mutants (Figure 9D) together with the observed colocalization of p-EphA2 with p-c-Cbl (Figure 4A) clearly suggested the need for tyrosine phosphorylation in the tyrosine kinase domain of EphA2 to facilitate its association with the c-Cbl TKB domain. The question of how these domain interactions aid EphA2 polyubiquitination needs additional detailed analysis. Nevertheless, since the SAM domain of EphA2 is known to help dimerization of EphA2 and thus activates its tyrosine kinase activity necessary for EphA2 functionality, c-Cbl TKB domain in the presence of the RING domain could be contributing to the association with the tyrosine kinase domain of dimerized EphA2 leading into its polyubiquitination.

The human EphA2 protein with 976 aa length has >30 lysine (K) residues in its cytosolic region (aa 559–976) spreading over kinase domain, SAM domain and PDZ domain. The minimal ubiquitination of truncated EphA2 as found in figure 9D may probably be due to the presence of lysine residue/s other than that specific mutated region. To determine the specific lysine residue/s undergoing polyubiquitination, extensive mutational and functional analysis are required which could be more intricate for a separate study and is beyond the scope of the current study.

However, since c-Cbl knockdown impairs EphA2 polyubiquitination (Figures 9 A and B) and EphA2 association with clathrin (Figures 10 A and B) and polyubiquitination appears to be one of the preferred sorting signals for clathrin mediated internalization [33], [58], our data suggest the role of c-Cbl induced polyubiquitination of EphA2 coordinated with EphA2 dependent modulation of KSHV-integrin interaction induced entry associated signaling as essential event for clathrin mediated KSHV entry.

Our studies show that c-Cbl mediated K63 type but not degradative K48 type polyubiquitination of EphA2 acts as an internalization signal for clathrin mediated virus entry. Sorting of ubiquitinated cargo from early to the late endosome and lysosome relies on endosomal sorting machinery known as ESCRT (endosomal sorting complex required for transport) [64]. Moreover, Ub may also be used as a sorting signal that gives proteins access to parts of the endocytic system without necessarily being degraded in lysosomes. For example, Ub might label receptors for transport to ‘signaling endosomes’, which would allow them to efficiently stimulate downstream signaling pathways. This could operate by allowing DUBs (deubiquitination enzymes) to intervene along the trafficking pathway to prevent efficient transport into lysosomes [65]. Therefore, even as cargoes are being sorted by ESCRTs, either last minute Ub conjugation with different E3 ligases or mutual regulation of Ub and DUBs (deubiquitination enzymes) for deubiquitination determines the fate of sorted molecules [66]. The sorting of specific cargoes can be influenced greatly by a particular polyUb chain, or that a polyUb chain can provide a set of powerful regulatory opportunities as evident from our study. Further studies are required to determine whether ESCRT and ESCRT-associated proteins play roles in KSHV infection.

Overall, our studies reveal EphA2 as a critical non-LR region associated molecule in fibroblast cells which coordinates the signals induced during interaction of KSHV with its entry receptors and regulates the endocytic events leading to clathrin mediated entry and productive infection (Figure 12). These results together with a similar role of EphA2 in LR-dependent KSHV entry in dermal endothelial cells [19], suggest a global or broad-spectrum effect of EphA2 in different cell types. These studies suggest that EphA2 and c-Cbl are promising therapeutic targets to control the initial stage of KSHV infection of endothelial, epithelial and fibroblast cells.

## Materials and Methods

### Cells and virus

Primary human foreskin fibroblast (HFF) cells (Clonetics, Walkersville, MD) and human embryonic kidney epithelial (HEK) 293 cells were grown as described before [11], [17], [67]. Induction of the KSHV lytic cycle in BCBL-1 cells, supernatant collection, and virus purification procedures were described previously [11]. KSHV DNA was extracted and copies were quantitated by real-time DNA PCR using primers amplifying the KSHV ORF 73 gene as described previously [25].

### Antibodies and reagents

Mouse anti-integrin α3β1 (Mab 1992), αVβ5 (Mab 2019Z), and αVβ3 (Mab 1976), and mouse anti-phosphotyrosine (p-tyr) (4G10 clone) antibodies were purchased from Chemicon International, Temecula, CA. Goat anti-β6 integrin antibody was purchased from Santa Cruz Biotechnology, CA. Mouse anti-c-Cbl, anti-phospho c-Cbl pY731 (phosphotyrosine) and clathrin antibodies were from BD Biosciences, San Diego, CA. Rabbit anti-caveolin-1 antibody, DAPI, Alexa 488 conjugated LysoTracker, Alexa 594 conjugated transferrin, Alexa 594 or 488 anti-rabbit and anti-mouse secondary antibodies were from Molecular Probes, Invitrogen, Carlsbad, CA. Protein A-Sepharose 6 MB and Protein G-Sepharose CL-4B were from Amersham Pharmacia Biotech, Piscataway, NJ. Anti-rabbit and anti-mouse antibodies linked to horse-radish peroxidase were from KPL Inc., Gaithersburg, MD. CD-71 hybridoma cell line was from American Type Culture Collection (ATCC), Manassas, VA. CD-71 Mab secreted in culture medium was purified by Protein-A-Sepharose affinity chromatography. Mouse monoclonal anti-KSHV gpK8.1A (4A4) antibody and rabbit monoclonal anti-KSHV gB were generated in our laboratory [68]. Rabbit anti-HA was from Zymed, Invitrogen,Carlsbad,CA and mouse anti-ubiquitin (P4D1) was from Santa Cruz, CA. Mouse anti-polyubiquitin FK-1, anti-K63 and -K48 monoclonal Abs were from Millipore, Temecula, CA. Rab5, EphA2, phospho-EphA2, phospho-Src, phospho-myosin IIA, myosin IIA, phospho-PI3-K, Epsin15 and AP-2 antibodies were obtained from Cell Signaling Technology, Danvers, MA. Heparin, other fine chemicals and buffers were purchased from Sigma, St Louis, MO.

### Generation of EphA2 shRNA transduced cells

HFF cells were transduced by lentiviruses encoding shEphA2 as described previously [19]. For validation of shRNA constructs, HEK293 cells were cotransfected with EphA2 expression plasmid (target) and shRNA lentiviral vector constructs. The construction and production of lentiviral gene transfer vectors were done as previously described [67]. Western blot analysis was performed to confirm the level of knockdown.

### Western blotting

Cells were lysed in RIPA buffer (15 mM NaCl, 1 mM MgCl2, 1 mM MnCl2, 2 mM CaCl2, 2 mM phenylmethylsulfonyl fluoride and protease and phosphatase inhibitor mixture) and incubated on a rocker at 4°C for 15 min. Lysates were normalized to equal amounts of protein, boiled in sample buffer, separated by 10% SDS-PAGE, transferred to nitrocellulose and probed with the indicated primary antibodies. Detection was by incubation with species-specific HRP-conjugated secondary antibodies. Immunoreactive bands were visualized by enhanced chemiluminescence (Pierce, Rockford, IL). The bands were scanned and quantitated using FluorChemFC2 and Alpha-Imager (Alpha Innotech Corporation, San Leonardo, CA).

### Immunoprecipitation

Clarified and pre-cleared two hundred (200) µg of cell lysates or one hundred and fifty (150) µg of Non-LR fractions were incubated for 2 h or overnight with immunoprecipitating antibody at 4°C, the resulting immune complexes were captured by Protein A or G-Sepharose and analyzed by Western blots using specific detection antibodies.

### Cell lysate preparation and ubiquitination of proteins

HFF cells either mock infected or infected with KSHV and HEK 293 cells transfected with various plasmid combinations followed by virus infection were lysed in 2% SDS lysis buffer (2% SDS, 150 mM NaCl, 10 mM Tris-HCl, pH 7.5, 2 mM EDTA, 10% glycerol, 1× protease inhibitor and 1× phosphatase inhibitor cocktail) and boiled for 5 min followed by sonication. Lysates were diluted 1∶10 in dilution buffer (10 mM Tris-HCl,pH 7.5, 150 mM NaCl, 2 mM EDTA, 1% Triton X-100), incubated at 4°C for 1 h with rotation and centrifuged at 20,000× g for 30 min.

Equal amounts of protein were used for immunoprecipitation. Immunoprecipitated proteins were washed with washing buffer (10 mM Tris-HCl, pH 7.5, 1M NaCl, 1 mM EDTA, 1% NP-40), boiled in SDS sample buffer, and separated on SDS-PAGE followed by western immunoblotting for ubiquitin specific antibodies.

### Lipid raft extraction and characterization

Lipid raft extraction was performed as per the manufacturer's protocol for the caveolae/rafts isolation kit (Sigma) based on non-detergent density gradient extraction of lipid rafts [69]. Briefly, HFF cells were lysed in 0.5 M sodium bicarbonate solution in water (500 mM sodium carbonate pH 11.0, 2 mM EDTA, 1 mM NaF, 1 mM orthovanadate, Sigma protease inhibitor cocktail). Cell lysates transferred into pre-cooled microfuge tubes were homogenized using a Dounce homogenizer (10 strokes) and sonicated for 10 secs. A discontinuous density gradient made of 5 layers of OptiPrep with different concentrations was prepared as described previously [18]. Two ml of gradient layer (35% OptiPrep) was placed at the bottom of the pre-cooled ultracentrifuge tube. Each OptiPrep layer was placed over the other using a Pasteur pipette. The tubes were ultracentrifuged at 45,000 rpm for 4 h using a Beckman SWI-55 rotor. One ml fractions were collected from the top of the centrifuge tube and pooled. Lipid raft containing fractions were characterized by the presence of caveolin-1 and non-lipid rafts were confirmed by the presence of CD-71 as described before [18].

### Measurement of KSHV entry by real-time DNA polymerase chain reaction (PCR)

HFF cells were infected with KSHV (30 DNA copies/cell) at 37°C for 1 h. For measuring KSHV entry, cells were washed with HBSS and partially bound uninternalized virus was removed with 0.25% trypsin-EDTA for 5 min at 37°C. Internalized KSHV DNA was quantitated by amplification of the ORF73 gene by real-time DNA PCR [25]. The KSHV ORF73 gene cloned in the pGEM-T vector (Promega) was used for the external standard. The cycle threshold (Ct) values were used to generate the standard curve and to calculate the relative copy numbers of viral DNA in the samples. Percentage inhibition was calculated by considering the ORF73 copy numbers in control shRNA transduced cells as 100%. A paired *t*-test was used between control and sh-RNA treated cells to obtain *p* values.

### Measurement of KSHV gene expression by real-time reverse transcription PCR (RT-PCR)

Total RNA was prepared from infected or uninfected cells using an RNeasy kit (QIAGEN) as described previously [25]. To quantitate viral gene expression, total RNA was subjected to ORF73 expression by real-time RT-PCR using gene specific primers and Taqman probes. The relative copy numbers of the transcripts were calculated from the standard curve using the Ct values of different dilutions of *in vitro*-transcribed transcripts. These values were normalized to GAPDH control reactions.

### Immunofluorescence microscopy

HFF cells seeded on 8 well chamber slides (Nalge Nunc International, Naperville, IL) were used. Infected and uninfected cells were fixed with 4% paraformaldehyde for 15 min, permeabilized with 0.2% Triton X-100, and blocked with Image-iTFX signal enhancer (Invitrogen). The cells were reacted with primary antibodies against the specific proteins, followed by fluorescent dye-conjugated secondary antibodies. For colocalization with transferrin, cells were incubated with fluid-phase marker Alexa 594 transferrin (35 µg/ml) at 37°C in the presence or absence of KSHV followed by immunostaining with the appropriate antibodies. Cells were imaged with a Nikon fluorescence microscope equipped with a Metamorph digital imaging system. Excitation and emission detection for each fluor was performed sequentially to avoid cross-talk. All experiments were performed at least three times.

Cololalization of mean pixel intensities are analyzed for three different fields with a minimum of 10 cells each with the metamorph pixel intensity calculator. The mean colocalization pixel intensities (for yellow color) are measured in arbitrary unit (a.u.) that are represented as percentage colocalization with respect to uninfected control in a graph and a paired t test is used to obtain the p values.

### Plasmids and transfection

Wild Type (pAM HA Cbl-Wt), RING mutant (pAM-HAC3HC4C5) and TKB mutant (pAM HA Cbl G306E) constructs of c-Cbl were generously provided by Dr. Hamid Band [70] (Eppley Institute for Cancer and Allied Diseases, University of Nebraska Medical Center). Wild Type EphA2 (pMyc-EphA2 Wt), Kinase Dead mutant (pMyc-EphA2 KD) and SAM mutant (pMyc-EphA2 SAM) of EphA2 constructs were a kind gift from Dr. Horonori Katoh (Laboratory of Molecular Neurolobiology, Graduate School of Biostudies, Kyoto University, Kyoto, Japan) [45]. HEK293 cells were transiently cotransfected with each of the HA-tagged c-Cbl wild type or mutant plasmids and Myc-tagged EphA2 wild type or mutant plasmids. Transfection was performed using 5 µg of plasmid DNA, Lipofectamine 2000 (Invitrogen) and Opti-MEM (Invitrogen) according to the manufacturer's instructions. After 48 h, the cells were serum starved and either mock infected or infected with KSHV (30 DNA copies/cell) for 5 min. Cell lysates were prepared for use in immunoprecipitation and immunoblotting studies.

### RNA interference using siRNA transfection

Transfection of primary HFF cells with siRNA was performed using the Neon transfection system (Invitrogen) according to the manufacturer's instructions. Briefly, subconfluent cells were harvested and washed once with 1×PBS and resuspended at a density of 1×10^7^ cells/ml in resuspension buffer R (provided by the company). 10 µl of this cell suspension was mixed with 100 pmol of siRNA and then microporated at room temperature using a single pulse of 1700 V for 20 ms. After microporation, cells were distributed into complete medium and placed at 37°C in a humidified 5% CO2 atmosphere. 72 hours post-transfection, cells were analyzed for knockdown efficiency by Western immunoblotting. All si-RNA oligonucleotides (siGenome SMARTpool) for c-Cbl and non-targeting siRNA pool no. 1 were purchased from Thermo Scientific (Catalog no. M-003003-02-0010 and D-001206-13-20, respectively).

### Statistical analysis

Data are expressed as means ± SD of at least three independent experiments (n≥3). In all tests, *p*<0.05 was considered statistically significant. Experiments in which p is <0.05 are marked with single asterisk and *p*<0.01 are marked with double *asterisk*.
